# Integrated multi-omics reveal the mechanisms of antioxidant, anti-inflammatory and aroma enhancement *in Scytosiphon lomentarius* via drying methods

**DOI:** 10.1016/j.fochx.2026.104146

**Published:** 2026-07-01

**Authors:** Haijiao Lin, Yang Song, Yuchen Sun, Qingyun He, Pu Xu, Chuhan Feng, Liya Duo, Siming Liu, Binbin Wei, Yuan Wang, Shaowei Yin

**Affiliations:** a School of Pharmacy, China Medical University, No.77 Puhe Road, Shenyang 110122, PR China; b Department of Anesthesiology, Shengjing Hospital of China Medical University, No.36 Sanhao Street, Shenyang, 110001, PR China; c Department of Gynecology and Obstetrics, Shengjing Hospital of China Medical University, No.36 Sanhao Street, Shenyang, 110001, PR China; d Department of Oncology, Shengjing Hospital of China Medical University, No.36 Sanhao Street, Shenyang, 110001, PR China

**Keywords:** *Scytosiphon lomentarius*, UPLC-ESI-QTOF/MS^E^, HS-GC–MS/MS, Drying methods, Inoxidizability, Anti-inflammation, Molecular docking

## Abstract

Drying is fundamental to *Scytosiphon lomentarius* processing. This study systematically evaluated how vacuum freeze-dried, microwave-dried, and hot-air dried affect its bioactivity and quality. We found that freeze-dried sample demonstrated superior rehydration capacity, notably enhanced antioxidant activity, and elevated bioactive compounds such as total phenols and flavonoids. This anti-inflammatory advantage was further validated in an LPS-induced RAW 264.7 cell model. Untargeted UPLC-ESI-QTOF/MS^E^ identified 135 non-volatile metabolites, with 18 differential metabolites as key to antioxidant activity variation. Molecular docking targeting iNOS revealed a marked increase in potential iNOS inhibitors in freeze-dried samples. Electronic nose analysis distinguished the three drying groups, while HS-GC–MS/MS tentatively identified 125 volatile compounds. Freeze-dried samples better retained Ionone, yielding caramel and creamy baked notes, while hot-air dried generated unpleasant dimethyl trisulfide. Collectively, freeze-dried proves optimal for *S. lomentarius* processing by best enhancing antioxidant capacity, anti-inflammatory activity, and characteristic volatile metabolites, providing a theoretical foundation for its high-quality production.

## Highlights

• First comprehensive evaluation of *S. lomentarius* across drying methods.
• Freeze-dried *S. lomentarius* exhibited the highest antioxidant activity.
• Freeze-dried identified more volatile compounds than all other drying methods.
• UPLC-ESI-QTOF/MS^E^ revealed 86 differential non-volatile markers.
• Hot-air and microwave drying may promote Glutamylglutamic acid in *S. lomentarius.*

## Graphical abstract

**Figure:**
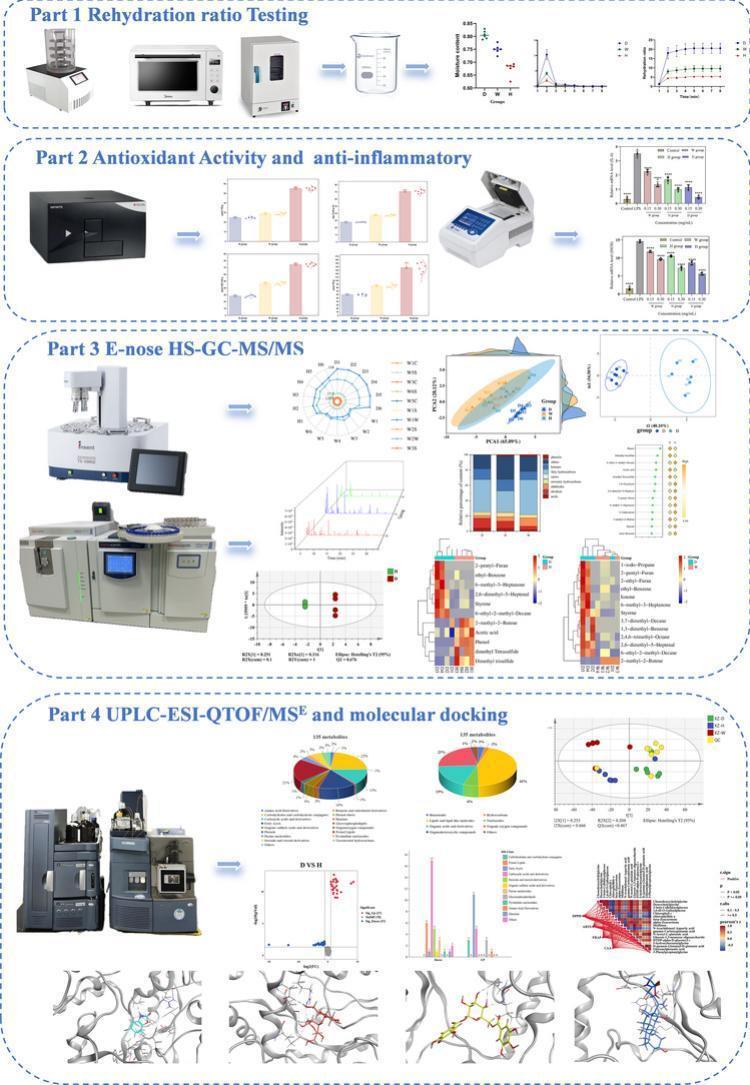

## 1 Introduction

*Scytosiphon lomentarius* (*S. lomentarius*) is a large brown alga widely distributed along the northern and southern coasts of China, and has long been consumed as a traditional food ingredient by coastal residents. Compared with common large seaweeds, *S. lomentarius* exhibits remarkable nutritional advantages: its protein content is higher than that of Porphyra (laver), and it is also rich in multiple dietary fibers and functional colloids, such as alginate and fucoidan (Cotas et al., 2024). In recent years, phlorotannins and fucoxanthin, which are specific to *S. lomentarius*, have been reported to exhibit significant antioxidant and anti-inflammatory activities (López-Hortas et al., 2023), distinguishing it from common edible seaweeds and establishing it as a promising candidate for functional food development and the extraction of natural bioactive compounds (Cmikova et al., 2025). However, the harvesting of *S. lomentarius* is characterized by pronounced seasonality, and its postharvest quality deteriorates rapidly, which necessitates the establishment of appropriate drying processing strategies to ensure its year-round stable supply and efficient utilization.

Drying is the most critical postharvest processing step in seaweed processing (Zia & Alibas, 2021). It extends shelf life by inhibiting enzymatic activity and microbial growth, and aims to maximize the retention of beneficial product properties. However, the heat and mass transfer processes also profoundly affect the retention and transformation of bioactive components (Li et al., 2025). For large brown algae, different drying methods exert particularly significant effects on phenolic compounds, fucoxanthin, and sulfated polysaccharides (Templonuevo et al., 2024). Previous studies have shown that vacuum freeze-dried can preserve phenolic acids and flavonoids to the greatest extent due to its low-temperature operation (Liu et al., 2022, Wang et al., 2022), albeit with high equipment costs. Hot-air dried is economical and practical, but heat treatment may induce isomerization and oxidative degradation of fucoxanthin (Wibowo et al., 2022). Microwave-dried exploits rapid volumetric heating to shorten drying time and achieve efficient heat and mass transfer, favorable quality retention, and lipid enrichment, while its low environmental and economic costs enhance its commercial scalability (Behera & Balasubramanian, 2021). These findings indicate that the choice of drying method plays a decisive role in quality regulation of algal materials.

Although the effects of drying methods on seaweed quality have been studied to some extent, systematic research on drying process optimization specifically for *S. lomentarius* remains a research gap. Therefore, it is essential to identify an optimal drying technique that enhances its antioxidant and anti-inflammatory activities. Analyzing the metabolite profiles of *S. lomentarius* under different drying procedures, combined with molecular docking studies, can help reveal the underlying mechanisms behind these quality variations. The findings of this study will provide valuable insights into the effects of common drying techniques on *S. lomentarius* quality, offering a solid scientific foundation and data support for selecting and optimizing high-quality, value-added drying strategies. This will further promote the development and application of *S. lomentarius* in functional foods and nutraceuticals.

## 2 Material and methods

### 2.1 Reagents and instruments

All chemicals and solvents used were analytical grade and required no further purification. Sodium nitrite (NaNO_2_, 7632-00-0), aluminium nitrate (AlNO_3_, 13473–90-0), ethanol (C_2_H_6_O, 64–17-5), hydrochloric acid (HCl, 7647-01-0), ferric chloride hexahydrate (FeCl_3_·6H_2_O, 10025–77-1), Rutin (C_27_H_30_O_16_, 153–18-4), Folin-Ciocalteu (C_6_H_6_O, 12111–13-6), gallic acid (C_7_H_6_O_5_, 149–91-7), sodium hydroxide (NaOH, 1310-73-2), acetic acid buffer were purchased from Yuanye Biotechnology Ltd. (Shanghai, China). The addition amount of organic solvents for all samples was kept to a minimum and consistent level, and it did not affect the measurement of antioxidant activity.

### 2.2 Processing and preparation of plant samples

*S. lomentarius* was purchased online and manually cleaned. The samples were randomly divided into three groups for drying using three distinct methods: vacuum freeze-dried (D), hot-air dried (H), and microwave dried (W). Vacuum freeze-dried was performed by evenly spreading the samples on trays in the drying chamber and drying them in a freeze-dryer at −80  °C for 11 h. Hot-air dried was carried out by uniformly placing the samples on shelves in drying oven and drying them at 40  °C for 6 h. Microwave dried was conducted by spreading the samples evenly on glass Petri dishes and drying them in a microwave oven at 800  W for 15 min. All the above drying conditions were based on previous studies and experimental gradient optimization (Fig. S1) (Yan et al., 2025). The dried samples were powdered using a grinder.

0.15 g of the dried powder of the samples was joined to 5 mL of 70% ethanol for extraction. Ultrasonic-assisted extraction was conducted for 30 min under optimized parameters (500 W, 40 kHz). Post-extraction, the mixtures were centrifuged at 12,000*g* for 30 min at 4 °C. The resulting supernatants were collected as *S. lomentarius* extract solutions. All extracts were stored at 4 °C in sealed containers to maintain stability. The resulting extracts were used for subsequent antioxidant activity assays (DPPH, ABTS and FRAP) and mass spectrometric analysis.

### 2.3 Rehydration ratio (RR1), rehydration rate (RR2), and moisture content (MC) after rehydration

The calculation methods for RR1, RR2, and MC refer to (Zhou et al., 2021). Six dried *S. lomentarius* samples, each weighing between 0.1 and 0.15 g, were prepared for each drying method and cut into small segments (1.5 cm) for analysis. A 100 mL beaker containing distilled water was placed in a water bath maintained at 25 °C. The dried samples were immersed in the beaker and removed at 1 min intervals. After each removal, the samples were blotted with filter paper and weighed. This procedure was repeated until a constant mass was attained, indicating that the dried samples had been fully saturated with water.

### 2.4 Antioxidant properties testing

Radical scavenging ability (DPPH, ABTS) and FRAP were adapted from our previous study with minor modifications (Wang et al., 2023). Detailed experimental procedures are described in Supplementary Data 1. The results of three antioxidant experiments were calculated based on a Trolox standard curve and are expressed as micromolar Trolox equivalents per gram (μmol  TE/g).

### 2.5 Cellular antioxidant activity (CAA) experiment

#### 2.5.1 Cell culture

Caco-2 cells were maintained in MEM containing 20% heat-inactivated FBS and 1% penicillin-streptomycin. The cells were maintained in an incubator at 37 °C with 5% CO₂. Upon reaching 80% confluence, cells were utilized for subsequent experiments.

#### 2.5.2 CAA assay

The CAA assay was performed according to the method described by (H. Lin et al., 2025) with modifications. In this study, the cells were plated at a density of 6 × 10^5^ cells per well in black, clear-bottom 96-well tissue culture plates and incubated at 37 °C for 24 to 48 h. To remove non-adherent and dead cells, the medium was aspirated and a PBS wash was performed post-incubation. Each well received 50 μL of antioxidant solutions and DCFH-DA (50 μM) in triplicate, respectively. After 20 min at 37  °C, cells underwent two PBS washes. Subsequently, 100 μL of AAPH solution (600 μM) was added to each well, and the plate was immediately transferred to a multi-mode microplate reader. The initial fluorescence was recorded, followed by measurements at 5 min intervals for 1 h, using 485/538 nm (ex/em), controls were defined as DCFH-DA^+^ AAPH without antioxidants, and blanks as DCFH-DA without AAPH or antioxidants. Quercetin standards were used to generate a calibration curve. The final results were expressed as μmol quercetin equivalents per 100 g dry weight (μmol QE/100 g).

### 2.6 Antioxidant capacity index (ACI)

ACI is a standard metric that is computationally determined. We calculated the ACI of different drying methods for *S. lomentarius* based on the calculation method of (Lin et al., 2022). Score = (sample score / best score) × 100%. The ACI was computed as the mean index score from four assays (DPPH, ABTS, FRAP, and CAA) to provide an integrated assessment of the antioxidant capacity.

### 2.7 TPC assay

TPC was determined following the method of (Shan et al., 2024) with slight adjustments. TPC was quantified by means of a gallic acid standard curve, with results reported as the gallic acid equivalents (GAE).

### 2.8 TFC assay

TFC was measured following a reported procedure (Liu et al., 2022) with slight adjustments. Quantification was based on a rutin standard curve, with results expressed as rutin equivalents (RE).

### 2.9 Anti-inflammatory assay

For the cell viability assay, RAW264.7 cells were seeded into 96-well plates at a density of 1 × 10^5^ cells/mL (100 μL per well) and allowed to adhere for 12 h. Subsequently, the cells were treated with various concentrations (150, 300, 450, and 600 μg/mL) of the freeze-dried (D), hot-air-dried (H), and microwave-dried (W) sample extracts for 24 h. After treatment, 15 μL of CCK-8 solution was added to each well, followed by incubation in the dark for 1 h. The absorbance of the supernatant in each well was then measured at 450 nm. Culture medium without *S. lomentarius* extract was used as the control.

Cell viability, nitric oxide (NO) content determination, and reverse transcription-quantitative polymerase chain reaction (RT-qPCR) analysis were performed according to the protocols previously established by our laboratory (Liu et al., 2024). The primer sequences used in this study are listed in Table S1.

### 2.10 Western blotting

RAW264.7 macrophages were stimulated with 1 μg/mL LPS for 24 h, and then treated with extracts of S. lomentarius prepared by different drying methods. The culture supernatant was aspirated, and the cells were washed three times with ice-cold PBS. RIPA lysis buffer containing 1 mM PMSF was added, and cells were lysed on ice for 30 min. The lysate was collected and centrifuged at 4 °C, and the supernatant was used as total protein. Protein concentration was determined using a BCA protein assay kit, and all samples were adjusted to equal concentrations before adding loading buffer and boiling for denaturation. Equal amounts of total protein were separated by SDS-PAGE and then transferred onto pre-activated PVDF membranes using a transfer apparatus. The PVDF membrane was blocked with 5% non-fat milk in TBST for 1.5 h at room temperature, and then incubated overnight at 4 °C with monoclonal antibodies against iNOS, IL-6, and the loading control β-actin, respectively. The next day, membranes were washed three times with TBST, followed by incubation with corresponding secondary antibodies at room temperature for 1 h, and washed three times again. Finally, protein bands were visualized using an ECL chemiluminescent detection reagent on a chemiluminescence imaging system, and the gray values of the bands were quantitatively analyzed using Image J software to compare the effects of different drying methods of *S. lomentarius* extracts on iNOS and IL-6 protein expression.

### 2.11 Untargeted non-volatile compounds analysis

The analysis was conducted using a Xevo G2-XS QTOF-MS and a Water ACQUITY UPLC system. The chromatographic separation used an ACQUITY UPLC™ C18 column (Waters, Milford, USA) maintained at 40 °C and an injection volume of 4 μL. A binary mobile phase system consisting of 0.1% formic acid in water (solvent A) and 0.1% formic acid in acetonitrile (solvent B) was used, with elution carried out at a flow rate of 0.35 mL/min over 22 min under the following gradient program: 0–2 min, 5% B; 2–15 min, directly increase to 95% B; 15–20 min, hold at 95% B; 20–22 min, return to 5% B; and re-equilibrate for 3 min. Metabolite identification followed the method of Liu et al. (Liu et al., 2025), with MS detection performed in both negative and positive ionization modes. The capillary voltage was set to 3.0 kV in positive mode and 2.0 kV in negative mode, while the sampling cone voltage was maintained at 40.0 V in both modes. Data were acquired in Continuum MS^E^ mode using a 0.2 s scan time across a mass range of 50–1200 Da.

### 2.12 Molecular docking analysis

Molecular docking studies on the chosen compounds were conducted with the MOE 2019.012 software. Chlorzoxazone (CLW), a co-crystallized native inhibitor, served as the reference. Compound structures were retrieved from PubChem (http://pubchem.ncbi.nlm.nih.gov/), and receptor proteins were sourced from the PDB (https://www.rcsb.org/). Because NO is the core inflammatory indicator and iNOS is the rate-limiting enzyme for NO production, iNOS was selected as the sole docking target to directly elucidate the NO inhibition mechanism, consistent with a previous report (Liu et al., 2024). To verify the docking protocol, CLW was re-docked into the iNOS binding site. The reliability of the method was assessed based on the resulting RMSD value, which was within the acceptable threshold (<2).

### 2.13 *E*-nose assay

The method of the e-nose reference (H. Lin et al., 2025) was adopted and slightly modified. The PEN3 (AIRSENSE, Germany), was an e-nose device containing ten distinct metal-oxide sensors forming a sensor array. The average value of the data from each sample was used for further analysis. Detailed information about the sensors was provided in Table S2.

### 2.14 Identification of non-volatile compounds

A customized internal library comprising 426 compounds was established using Progenesis SDF Studio. Raw data acquired from UPLC-ESI-QTOF/MS^E^ analysis via Masslynx software were processed in Progenesis QI 2.3 (Waters, UK) for untargeted metabolite identification against the internal library and HMDB database. Identification was via the following criteria, listed in decreasing order of significance: mass error tolerances for precursor and fragment ions (≤10 ppm for both), isotope similarity (>80%), identification score (>30), and software-generated fragment score. All mass spectra were verified to be derived from a single compound, and identifications with over 80% confidence in each sample were retained for initial assessment.

### 2.15 Untargeted volatile compounds analysis

Volatile compounds were analyzed by HS-GC–MS/MS utilizing an Agilent 7890B gas chromatograph coupled to a Thermo Orbitrap Exploris GC mass spectrometer. A 1.5 g sample of powder was placed into a 10 mL headspace vial and subjected to incubation at 100  °C for 10 min. Subsequently, it was subjected to 4 min of desorption. Separation was performed on a TG-5MS quartz capillary column (30 m × 0.25 mm × 0.25 μm) with helium (99.999%) as the carrier gas at a constant flow rate of 1 mL/min. Key GC parameters included: vial pressure 90 kPa, quantitative loop pressure 70 kPa, loop temperature 95 °C, and inlet temperature 200  °C. The column temperature program was set as follows: 40  °C for 2 min, increased to 90 °C at 8 °C/min and held for 2 min, raised to 180 °C at 6  °C/min and held for 2 min, then to 260 °C at 15 °C/min and held for 2 min, followed by a 3 min run under a 10:1 split ratio. The transfer line and column temperatures were maintained at 230 °C. MS detection was conducted in electron impact (EI) mode with an ion source temperature of 230 °C, a mass range of 30–600 Da, and a resolution of 30,000. Compounds were preliminarily identified by matching mass spectra against the NIST 17.0 database (match >80%) and quantified as relative peak area percentages (Z. Song et al., 2025).

### 2.16 Statistical analysis

Multivariate statistical analysis was performed with Simca-p 14.1 (SIMCA Imola s.c., Italy), while Origin 2021 (Origin Lab Corporation, Northampton, MA, USA) and ChiPlot (https://www.chiplot.online/.) were used to draw the graphs. Statistical analysis was performed using SPSS 18.0, including one-way ANOVA and tests for homogeneity of variance. Significant differences were determined by Duncan's test (*P* < 0.05), and data are presented as mean ± SD.

## 3 Results and discussion

### 3.1 The analysis of RR1, RR2, and MC after rehydrating

**Fig. 1:**
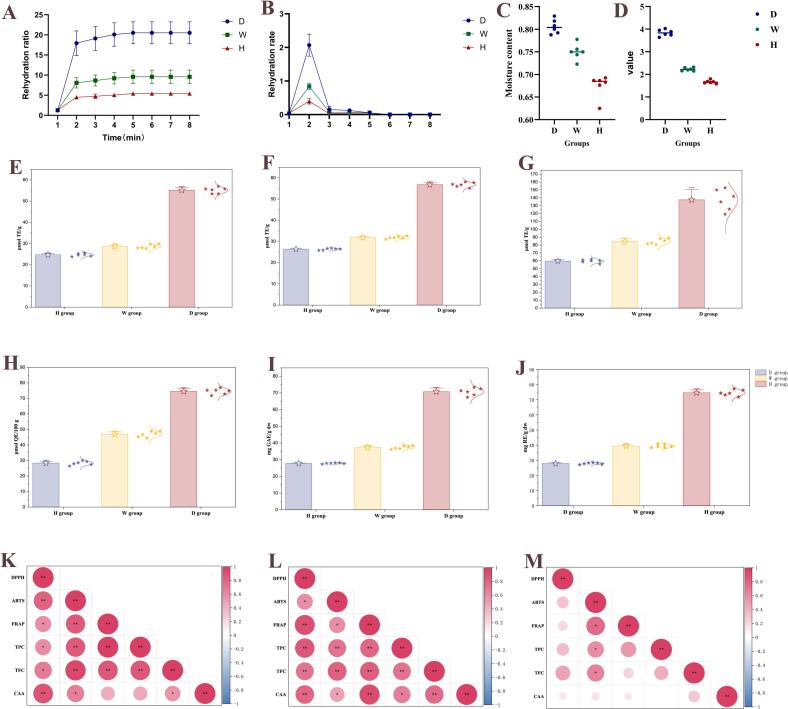
Rehydration ratio (A), rehydration rate (B), and moisture content (C) of dried *S. lomentarius*; ACI score (D); Antioxidant activity of dried *S. lomentarius*: DPPH (E), ABTS (F), FRAP (G), CAA (H); TPC and TFC of dried *S. lomentarius*; Correlation analysis of antioxidant activity in the freeze-dried group (K), hot-air-dried group (L); the microwave-dried group (M). (* Correlation is significant at the *P* < 0.05; ** Correlation is significant at the *P* < 0.01; *** Correlation is significant at the *P* < 0.001, *n* = 3. D represents vacuum freeze-dried, W represents microwave dried and H represents hot-air dried).

Rehydration capacity is a core quality indicator affecting the drying and processing performance of algae. The rehydration rate, rehydration ratio, and moisture content after rehydration are shown in Fig. 1A, B, and C, respectively. The freeze-dried group exhibited the highest RR1, while the hot-air-dried group showed the lowest. RR2 results revealed consistent trends across all three groups, with maximum rates occurring within 2 min, followed by gradual deceleration until reaching zero. The RR2 trend mirrored the RR1 pattern, with the freeze-dried group demonstrating superior performance. MC analysis indicated the freeze-dried group recovered to approximately 80%, the microwave-dried group to 75%, and the hot-air-dried group to 68%. This may stem from the freeze-dried group's porous microstructure potentially enhancing water uptake during rehydration, thereby improving hydrophilic properties. In contrast, the hot-air-dried and microwave-dried groups underwent mechanical alterations in texture, with compressed tissue structures limiting cellular capacity to capture moisture during rehydration (Yan et al., 2025). These findings suggest freeze-dried possesses rapid rehydration capability and holds greater potential for achieving higher rehydration ratio.

### 3.2 The analysis of antioxidant activity

#### 3.2.1 The analysis of DPPH, ABTS and FRAP

The specific data results of DPPH, ABTS and FRAP are presented in Table S3. This study utilized ABTS and DPPH radical scavenging assays to further study the potential effects on the antioxidant activity of *S. lomentarius*. As shown in Fig. 1E and F, the freeze-dried group exhibited the strongest radical scavenging capacity, while the hot-air-dried group demonstrated the weakest. The microwave-dried group showed stronger radical scavenging capacity than the hot-air-dried group. In the DPPH assay, the freeze-dried group ranged from 53.40 ± 0.17 to 56.96 ± 0.32 μmol TE/g. In the ABTS assay, the freeze-dried group ranged from 55.09 ± 0.61 to 58.09 ± 0.13 μmol TE/g.

The FRAP assay measures the reducing power of a sample by assessing its capacity to reduce Fe^3+^ to Fe^2+^. The results align with the trends observed in the previous two radical scavenging experiments, as shown in Fig. 1G. The freeze-dried group ranged from 119.16 ± 0.52 to 152.75 ± 0.23 μmol TE/g. Based on the FRAP measurement principle, we hypothesize that the Maillard reaction generates strong reducing components, thereby significantly enhancing FRAP values. We conclude that the freeze-dried group exhibited optimal antioxidant activity, closely related to its drying process under low-temperature and vacuum conditions.

#### 3.2.2 CAA analysis

The growing focus on dietary antioxidants has created a need for methods that can accurately predict their antioxidant capacity in vivo. Cell assays are regarded as a more suitable middle ground between in vitro chemical tests and in vivo studies (Martinelli et al., 2021). CAA results are shown in Fig. 1H, with group-specific values listed in Table S3. The freeze-dried group exhibited CAA values ranging from 71.77 ± 1.26 to 76.92 ± 1.07 μmol QE/100 g, while the microwave-dried group showed values of 44.5 ± 1.5 to 48.9 ± 0.48 μmol QE/100 g. The hot-air-dried group exhibited values ranging from 26.36 ± 0.98 to 29.97 ± 1.1 μmol QE/100 g. Notably, this trend clearly aligns with the antioxidant capacity patterns observed in the DPPH, ABTS, and FRAP assays described earlier.

#### 3.2.3 ACI analysis

The ACI is defined as the equal-weighted combination of multiple assays, fully representing overall antioxidant capacity. As shown in Fig. 1D, the freeze-dried group exhibited a higher ACI than both the microwave-dried and hot-air-dried groups. Specifically, the freeze-dried group's ACI was 1.5 times that of the microwave-dried group and 2.2 times that of the hot-air-dried group. This result further confirms that freeze-dried *S. lomentarius* exhibits superior antioxidant capacity compared to both the microwave-dried and hot-air-dried groups.

### 3.3 TPC analysis

Drying is an essential food preservation technique, and the retention of phenolic content is crucial. Phenolic components also exhibit significant differences among samples subjected to different treatments. According to Fig. 1I, the trends of the three groups were the same as the antioxidant properties. Detailed data are presented in Table S3. Low temperatures inhibit the oxidative degradation of phenolic compounds (Fan et al., 2025), while higher temperatures accelerate the degradation of heat-sensitive bioactive compounds. This aligns with our findings that freeze-dried *S. lomentarius* extracts exhibited the highest TPC content.

### 3.4 TFC analysis

Flavonoids are phytochemical compounds found in many plants, including fruits, vegetables, and leaves, and possess certain medicinal properties such as anticancer, antioxidant, anti-inflammatory, and antiviral characteristics. As shown in Fig. 1J, the trend in TFC content aligns with the aforementioned antioxidant activity trend. The freeze-dried group ranged from 71.79 ± 0.72 to 77.21 ± 0.70 mg RE/g dw. Detailed data are presented in Table S3. Flavonoids with abundant phenolic hydroxyl groups are prone to degradation and oxidation at high temperatures, especially those containing aldehyde groups, where heat induces structural changes and losses. This likely explains the lower TFC observed in microwave-dried and hot-air-dried samples.

### 3.5 Correlation analysis

Pearson correlation analysis was performed on DPPH, ABTS, FRAP, CAA, TPC, and TFC, to further explore the relationship between antioxidant capacity and TPC, TFC. The correlation plots for the freeze-dried group (Fig. 1K), the hot-air-dried group (Fig. 1L) and the microwave-dried group (Fig. 1M) reveal a strong positive correlation between TPC and TFC across all three groups. Detailed correlation data in Table S4 show Pearson coefficients exceeding 0.72 between TPC and TFC, indicating their biological activity may stem from structurally or functionally similar compounds. Correlation results between antioxidant activity (DPPH, ABTS, FRAP, and CAA) and TPC, TFC also showed positive correlations: Pearson coefficients exceeded 0.448 in the freeze-dried group, exceeded 0.675 in the microwave group, and exceeded 0.540 in the hot-air-dried group. This suggested that the antioxidant capacity of *S. lomentarius* may primarily originate from phenolic and flavonoid compounds, necessitating further metabolite research using untargeted metabolomics.

### 3.6 Anti-inflammatory activity analysis

#### 3.6.1 Effects of three drying methods on the viability of RAW264.7 cells

**Fig. 2:**
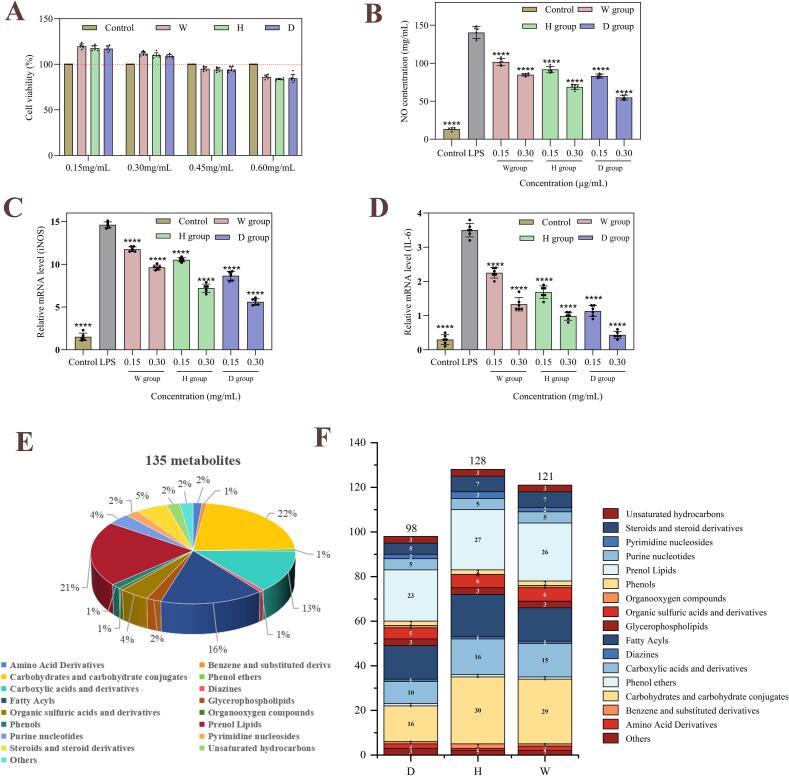
Anti-inflammatory effect of *S. lomentarius* under different drying treatments. Cell viability of extract of *S. lomentarius* on RAW264.7 macrophages (A); Inhibition of freeze-dried, hot-air-dried and microwave-dried on LPS-induced NO production (B); Inhibition of mRNA of iNOS (C) and IL-6 induced (D); 3D pie chart classifying 135 metabolites (E); stacked bar chart of metabolites from three drying methods (F). (D represents vacuum freeze-dried, W represents microwave dried and H represents hot-air dried; ****, *P* < 0.0001, *n* = 3).

A RAW 264.7 macrophage inflammation model was established to assess the effects of different drying methods on the anti-inflammatory activity of *S. lomentarius*. Initially, the impact of *S. lomentarius* extracts on cell viability was assessed using the CCK-8 assay. As shown in Fig. 2A, treatment with extracts obtained by different drying methods at concentrations ranging from 0.15 to 0.6 mg/mL did not inhibit the growth of RAW 264.7 cells, with viability exceeding 100%, and no cytotoxicity was observed at lower concentrations. Therefore, for subsequent experiments, the three groups of *S. lomentarius* extracts were tested at concentrations of 0.15 mg/mL and 0.30 mg/mL, with six replicates per group.

#### 3.6.2 The three drying methods inhibited NO production by suppressing RAW264.7 macrophages

NO is a biological free radical that acts as a neurotransmitter involved in regulating various biological processes, including mediating apoptosis and the production of inflammatory cytokines, and is commonly used as an inflammatory marker (Cui et al., 2025). As shown in Fig. 2B, compared to the blank group, NO secretion increased significantly after LPS stimulation (*P* < 0.0001), confirming the successful establishment of the inflammation model. At a concentration of 0.15 mg/mL, *S. lomentarius* subjected to different drying methods significantly inhibited LPS-stimulated NO secretion in RAW 264.7 cells. Samples treated with different drying methods significantly promoted NO secretion at concentrations of 0.15 mg/mL and 0.30 mg/mL (*P* < 0.0001), with the freeze-dried group showing the most pronounced effect—recording levels of 83.17 ± 2.63 μg/mL and 54.83 ± 3.13 μg/mL at 0.15 mg/mL and 0.30 mg/mL, respectively—followed by the hot-air-dried and microwave-dried groups. *S. lomentarius* samples treated with the three drying methods were able to prevent macrophage apoptosis induced by excessive NO. Therefore, different processing methods may influence immunostimulatory activity by altering the properties and structural characteristics of *S. lomentarius*.

#### 3.6.3 The three drying methods inhibited the secretion of iNOS and IL-6 by suppressing the activation of RAW264.7 macrophages

Inflammation is a self-protective mechanism of the body, involving the regulation of multiple cytokines. Among them, inducible nitric oxide synthase (iNOS) and interleukin-6 (IL-6) are common pro-inflammatory factors and are often used as inflammatory markers. As shown in Fig. 2 C and D, the secretion of both pro-inflammatory factors increased significantly after stimulation with LPS alone (*P* < 0.0001). In the analysis of iNOS expression, RAW264.7 macrophages treated with freeze-dried (D), hot-air-dried (H), or microwave-dried (W) samples exhibited a dose-dependent reduction in iNOS mRNA expression compared to those treated with LPS only, with all treatments showing significant inhibitory effects (*P* < 0.0001). In the analysis of IL-6, the results followed a similar trend to that of iNOS, with the inhibitory effect ranking as follows: D > H > W. This pattern suggests that as the drying method changed, the reduction in LPS-induced IL-6 mRNA expression gradually weakened, indicating a corresponding decline in anti-inflammatory activity. These findings demonstrate that *S. lomentarius* extracts possess potential anti-inflammatory activity. However, heat-involved drying processes may lead to the degradation or loss of active anti-inflammatory compounds. Similar results have been reported in previous studies, which found that freeze-dried better preserves the compounds responsible for inhibiting specific pro-inflammatory cytokines (Yang et al., 2025).

#### 3.6.4 Western blot detection of iNOS and IL-6 protein expression in response to different drying methods of *S. Lomentarius* extracts

To compare the inhibitory effects of *S. lomentarius* samples processed by different drying methods on LPS-induced inflammation in RAW264.7 cells, Western blot was performed to detect the protein expression levels of the pro-inflammatory factors iNOS and IL-6 (Fig. S2A). Compared with the Control group, the LPS-only stimulated group showed extremely significant increases in both iNOS and IL-6 expression (*P* < 0.0001), indicating that the inflammatory model was successfully established (Fig. S2 B and C). Compared with the LPS group, treatment with W, H, and D resulted in varying degrees of downregulation of iNOS and IL-6 expression. Specifically, for iNOS, the freeze-dried group (D) showed the faintest band, indicating the most pronounced inhibitory effect, followed by the oven-dried group (H) with slightly stronger band intensity, while the microwave-treated group (W) exhibited the strongest band intensity and thus the weakest inhibition. These results suggest that all three treatments possess anti-inflammatory activity, with IL-6 expression following a trend similar to that of iNOS. However, differences in anti-inflammatory potency were observed among the three drying methods, with the freeze-dried sample exerting the strongest effect in this model.

### 3.7 Identification of non-volatile compounds

Using UPLC-ESI-QTOF/MS^E^ technology for analysis, 135 metabolites were found across different drying treatments. Detailed information on these compounds, including their Name, HMDB ID, Formula, Super class, Class, and Score, is summarized in Supplementary Data 2. The 135 identified metabolites were categorized into 8 super classes (Fig. S3A) and 17 classes (Fig. 2E). The 17 classes consisted of 30 carbohydrates and carbohydrate conjugates (22.22%), 28 prenol lipids (20.74%), 21 fatty acyls (15.56%), 18 carboxylic acids and derivatives (13.33%), 7 Steroid derivatives and steroids (5.19%), 6 Organic sulfuric acids and derivatives (4.44%), 5 Purine nucleotides (3.70%), 3 Unsaturated hydrocarbons (2.22%), 3 Pyrimidine nucleosides (2.22%), 3 Glycerophospholipids (2.22%), 2 types of Phenols (1.48%), 2 types of Amino Acid Derivatives (1.48%), 1 type of Organooxygen compounds (0.74%), 1 type of Diazines (0.74%), 1 type of Phenol ethers (0.74%), 1 type of Benzene and substituted derivatives (0.74%), and 3 types of Others (2.22%). Furthermore, grouping the 135 metabolites into bar-column categories (Fig. 2F) clearly reveals differences in the number of identified metabolites across drying treatments: 98 metabolites were identified in the freeze-dried group, 128 in the hot-air-dried group, and 121 in the microwave-dried group.

### 3.8 Multivariate statistical analysis of non-volatile compounds

**Fig. 3:**
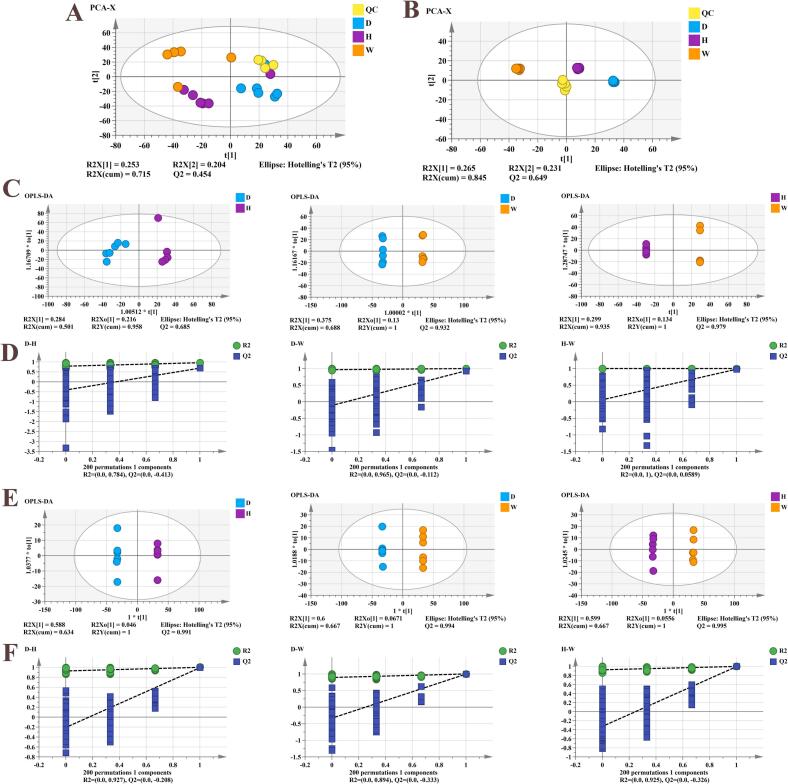
PCA plot in positive ion mode (A); PCA plot in negative ion mode (B); OPLS-DA scoreplot (C) and permutation plot (D) in positive ion mode based on UPLC-ESI-QTOF/MS^E^: freeze-dried group vs. hot-air-dried group, freeze-dried group vs. microwave-dried group, hot-air-dried group vs. microwave-dried group; OPLS-DA scoreplot (E) and permutation plot (F) in positive ion mode: freeze-dried group vs. hot-air-dried group, freeze-dried group vs. microwave group, hot-air-dried group vs. microwave group.

PCA is an unsupervised clustering method used to obtain an overview of *S. lomentarius* metabolite variations without prior knowledge of the dataset. Following preprocessing of ESI^+^ and ESI^−^ mode metabolomics data, it serves to reveal metabolic variation patterns across all samples. In the positive ion mode, the first two principal components (PC1 and PC2) accounted for 25.3% and 20.4% of variance, respectively (Fig. 3A). In the negative ion mode, they accounted for 26.5% and 23.1% of variance, respectively (Fig. 3B). The results revealed clustering of samples dried using the same method, while samples dried differently remained separated, indicating significant differences between drying methods in the principal component space. Furthermore, in the PCA plots, QC samples clustered tightly and were positioned near the origin, demonstrating experimental stability and reproducibility, and confirming the accuracy of both positive and negative ion models.

To further validate the effectiveness of PCA clustering and screen for compounds with distinct characteristics, an OPLS-DA supervised model was constructed. In the positive ion mode, as shown in (Fig. 3C) and negative ion mode (Fig. 3D), result displayed that all three processing methods of Scytosiphon lomentarius exhibit significant separation trends, highly consistent with PCA analysis outcomes. Concurrently, 200 permutation tests validated the OPLS-DA model's robust predictive capability and reliability. In positive ion mode (Fig. 3E) and negative ion mode (Fig. 3F), the groups were: freeze-dried vs. hot-air-dried, freeze-dried vs. microwave-dried, and hot-air-dried vs. microwave-dried. No overfitting was observed in either mode, confirming the model's excellent predictive capability and reliability.

### 3.9 Identification of differentiated compounds

**Fig. 4:**
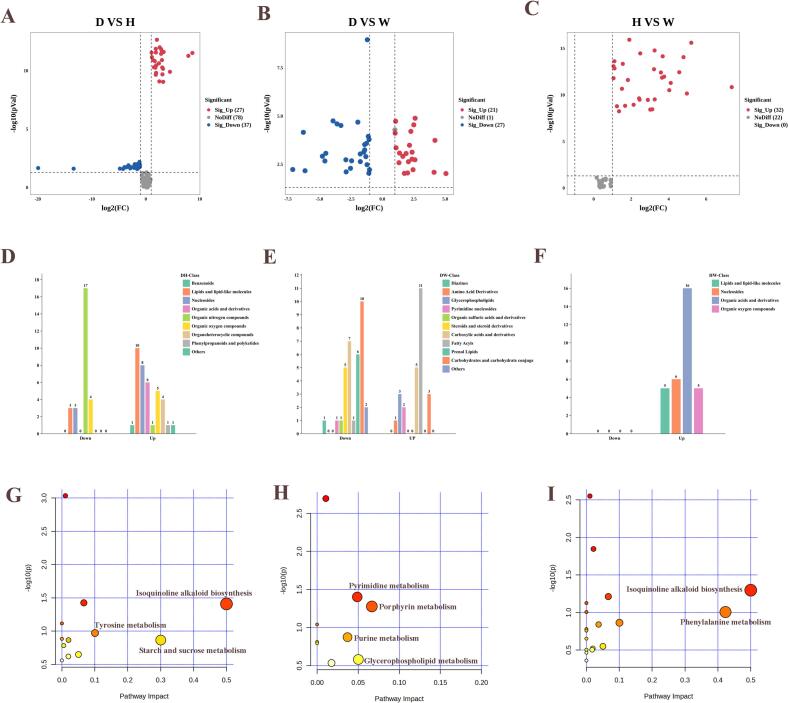
Volcano plot of differential metabolites between freeze-dried vs. hot-air-dried groups (A), freeze-dried vs. microwave-dried groups (B), hot-air-dried group vs. microwave-dried group (C), classification diagram of up and down regulated differential metabolites between freeze-dried vs. hot-air-dried groups (D), freeze-dried vs. microwave-dried groups (E), hot-air-dried group vs. microwave-dried group (F); analysis of metabolic pathways of *S. lomentarius* after freeze-dried (G), analysis of metabolic pathways of *S. lomentarius* after hot-air-dried (H), analysis of metabolic pathways of *S. lomentarius* after microwave-dried (I).

To further analyze metabolites, we screened a total of 86 differentially expressed metabolites according to VIP >1, fold changes ≥2 or ≤ 0.5, and *p*-values <0.05. Among these, the freeze-dried vs. hot-air-dried groups yielded 64 differentially expressed metabolites (Fig. 4A), comprising 27 up-regulated and 37 down regulated. The classification of up and down regulated metabolites was shown in Fig. 4D. The freeze-dried vs. microwave groups yielded 48 differentially expressed metabolites (Fig. 4B), comprising 21 up regulated and 27 down regulated metabolites. The classification of up and down regulated metabolites shown in Fig. 4E. The hot-air-dried vs. microwave groups yielded 32 differentially expressed metabolites (Fig. 4C), all down regulated. The classification of these metabolites is shown in Fig. 4F. The classification of 86 differential metabolites is shown in Fig. S1 B. Organic acids and derivatives constituted the largest group with 35 metabolites, accounting for 40.70% of all differential substances, followed by lipids and lipid-like molecules with 16 metabolites. The differential accumulation and degradation of metabolites in the three dried *S. lomentarius* samples may be related to temperature, enzyme activity, and drying time. The identified differential metabolites serve as diagnostic biomarkers for different drying methods of *S. lomentarius* and are key determinants of its quality attributes. They hold dual significance, particularly regarding sensory characteristics and pharmacological efficacy. Characterizing these metabolites provides in-depth theoretical support for processing, production, drying selection, and resource utilization.

#### 3.9.1 Organic acids and derivatives

In this study, the most abundant differential compounds were organic acids and derivatives (Fig. S3B). Organic acids, especially carboxylic acids, are generally heat-sensitive and thus prone to degradation during thermal processes like drying. Research (Kraseasintra et al., 2023) investigated L-tyrosine extraction from *A. platensis* biomass residue utilizing two drying methods: freeze-dried and hot-air dried. The results showed that L-tyrosine content was highest in the hot-air dried group, consistent with our findings. The highest tyrosine content was identified in the hot-air-dried group, followed by microwave dried, with freeze-dried yielding the lowest content. Glutamic acid (Glu) is considered one of the umami amino acids most closely associated with flavor characteristics in seafood. In our study, Glutamylglutamic acid was not detected in the freeze-dried group but appeared in the other two drying methods. This finding was also validated when investigating the effects of different drying methods on edible roots and tubers, confirming that different drying techniques indeed cause changes of Glu (Q. Wang, Zhang, Huang, Shi, Zhang, Jiang, et al., 2022). This indicates that pre-dried *S. lomentarius* with microwave or hot-air dried before cooking can produce functional foods rich in Glu. This method for preparing Glu-rich foods is rapid, effective, simple, and inexpensive. In addition, Aspartic acid (Asp) and Glu are considered the two umami amino acids most closely associated with flavor characteristics in seafood.

#### 3.9.2 Lipids and lipid-like molecules

In this study, a relatively large number of differential compounds were lipids and lipid-like molecules (Fig. S3B). Lipids and lipid-like molecules include steroids and steroid derivatives. Alpha-zeacarotene was not detected in the freeze-dried group but was detected in both the microwave-dried and hot-air-dried groups. This indicates that the retention levels of alpha-zeacarotene vary under different processing methods, consistent with the findings of the study on (Y. Song et al., 2021). Fucoxanthin is a marine lutein-type carotenoid with health-promoting properties, including anti-cancer, anti-inflammatory, anti-obesity and anti-diabetic effects. It has garnered significant attention for its potential in cancer prevention, weight management, blood sugar regulation, lipid-lowering, and antioxidant activities (Chinnappan et al., 2024). Furthermore, the hydroxyl group and conjugated double bonds in fucoxanthin's molecular structure support direct radical scavenging. The lipophilic nature of fucoxanthin enables its intracellular accumulation and activation of the Nrf2/Keap1/ARE pathway. By elevating GSH levels and NQO1 expression, it enhances endogenous antioxidant capacity, providing experimental evidence for its potential development as a functional ingredient targeting antioxidant-related diseases (Pruccoli et al., 2024).

### 3.10 KEGG pathway enrichment analysis

MetaboAnalyst with the KEGG database was used to uncover the molecular mechanisms behind metabolite alterations in *S. lomentarius* caused by different drying techniques. Using pathway libraries and *Arabidopsis thaliana* as references, we identified KEGG compounds. In the enrichment analysis, the vertical axis represents *p*-values, where higher values indicate a greater number of metabolites involved in the pathway. The horizontal axis corresponds to pathway topology analysis, where higher impact values signify a more significant influence of the identified metabolites on the pathway. Results indicate that metabolic pathways associated with differential metabolites freeze-dried include Starch and sucrose metabolism, Isoquinoline alkaloid biosynthesis and Tyrosine metabolism (Fig. 4G, Table S5), suggesting that the treatment conditions may have influenced the synthesis of secondary metabolites and energy metabolism. Specifically, during freeze-dried, the formation and sublimation of ice crystals likely imposed specific stresses on cellular structures, which could strongly activate or inhibit these metabolic pathways. In the hot-air dried group, pathways such as Purine metabolism, Pyrimidine metabolism, Porphyrin metabolism, and Glycerophospholipid metabolism were notably associated (Fig. 4H, Table S6). This pattern may be attributed to the sustained and relatively gentle thermal dehydration process, which generally-yet non-specifically-affected fundamental metabolic functions. For the microwave-treated group, pathways including Isoquinoline alkaloid biosynthesis and Phenylalanine metabolism were prominently enriched (Fig. 4I, Table S7). The rapid internal heating characteristic of microwave treatment may have instantaneously altered enzyme activity or substrate availability, thereby specifically interfering with the biosynthesis of such secondary metabolites. In summary, all three drying methods significantly altered the metabolic profile of the samples, albeit through distinct modes of influence. This study reveals, at the metabolic pathway level, that freeze-dried induced intense perturbations in a few key pathways, particularly those related to secondary metabolism and energy metabolism. Hot-air dried resulted in widespread yet moderate alterations across fundamental metabolic networks, while microwave dried exerted highly focused effects, primarily on phenylpropanoid metabolism. These findings provide critical theoretical insights for the precise selection of drying processes aimed at preserving specific bioactive components or functional properties.

### 3.11 *E*-nose analysis

**Fig. 5:**
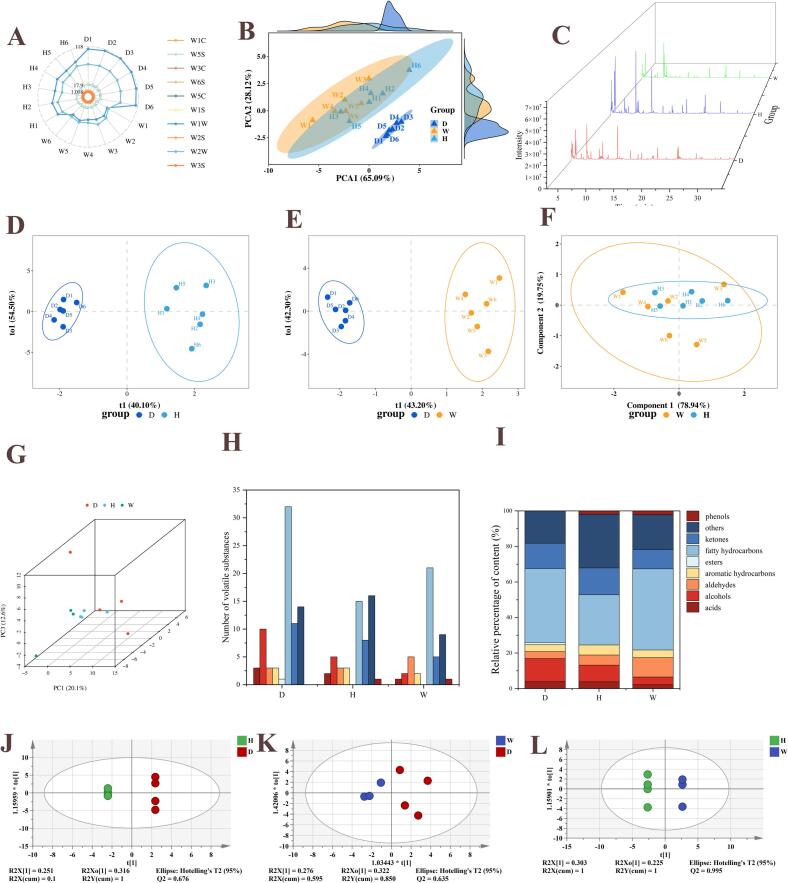
Analysis of e-nose radar diagrams (A); analysis of e-nose PCA (B); The TIC based on HS-GC–MS/MS (C); analysis of e-nose OPLS-DA: freeze-dried vs. hot-air-dried (D), freeze-dried vs. microwave-dried (E), hot-air-dried vs. microwave-dried (F); 3D PCA plot of volatile compounds (G); Bar chart grouping volatile compounds (H); Accumulated bar chart grouping volatile compounds (I); OPLS-DA scoreplots of volatile compounds: freeze-dried vs. hot-air-dried (J), freeze-dried vs. microwave-dried (K), hot-air-dried vs. microwave-dried (L).

The overall flavor profile of *S. lomentarius* under different drying methods was analyzed using an electronic nose, and the radar chart (Fig. 5A) illustrates the sensitivity toward various sensors. Overall, sensors W1W and W2W exhibited relatively high values in this study, indicating a higher content and greater sensitivity toward sulfides and organic sulfides in *S. lomentarius* samples subjected to different drying methods. Furthermore, the sensitivity of the sensors varied among the drying methods, following the order: freeze-dried (D) > hot-air dried (H) > microwave dried (W).

PCA was applied to classify the samples based on the intensity of electronic nose signals (Fig. 5B), highlighting differences in volatile components. The PC1 (65.09%) and PC2 (28.12%) cumulatively accounted for 93.21% (>90.00%) of the total variance, effectively capturing nearly all sample information and representing distinctions among the samples. To further identify specific sensor response differences among the three drying methods for *S. lomentarius*, unsupervised OPLS-DA was performed on the sensor response values. As shown in Fig. 5D, E and F, the separation efficiencies for D vs. W, D vs. H, and H vs. W were 85.50%, 94.60%, and 98.69%, respectively. These results demonstrate that the electronic nose method can effectively differentiate *S. lomentarius* samples processed by different drying techniques. For an in-depth investigation, HS-GC–MS/MS was further applied to generally, systematically, and accurately compare and analyze odor differences among *S. lomentarius* samples at the molecular level.

### 3.12 Identification of volatile compounds

To investigate changes in volatile metabolites of *S. lomentarius* during freeze dried, microwave dried, and hot-air dried we performed preliminary analysis and identification using HS-GC–MS/MS, with the total ion chromatogram (TIC) analysis shown in Fig. 5C. Based on match scores exceeding 80%, 125 volatile compounds were preliminarily identified. Specific compounds are listed in Supplementary Data 3, including 16 ketones, 15 fatty hydrocarbons, 9 aldehydes, 4 acids, 3 aromatic hydrocarbons, 1 phenol, 1 ester, and 25 others. Fig. 5H displays the number of volatile metabolites in each group, while Fig. 5I shows the relative percentage of volatile compound content. In terms of total quantity, freeze-dried samples yielded more detectable volatile compounds than hot-air-dried or microwave-dried samples. The freeze-dried group preliminarily identified 77 volatile components, the hot-air-dried group identified 53 volatile components, while the microwave group identified fewer volatile components than both groups, with only 46 volatile components detected. This may be attributed to the fact that as drying time increases during hot-air dried, the number of physical and chemical reactions occurring in the sample rises, thereby affecting the quantity of volatile organic compounds. Conversely, microwave dried promotes the polymerization and condensation of volatile substances, similarly reducing their quantity. The lowest temperatures employed during freeze-dried most effectively inhibit the myriad physical and chemical reactions occurring during the drying phase. This process preserves the original compounds in the algae samples, offering particular advantages for products of high economic value and rich flavor.

### 3.13 Identification of volatile compound variants

**Fig. 6:**
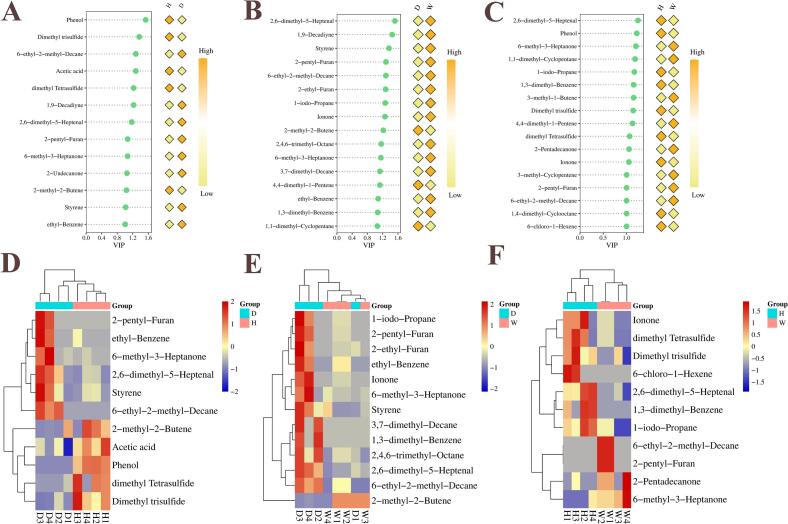
Volatile compounds' VIP plots: freeze-dried vs. hot-air-dried (A), freeze-dried vs. microwave-dried (B), hot-air-dried vs. microwave-dried (C); Cluster analysis of volatile compounds: freeze-dried vs. hot-air-dried (D), freeze-dried vs. microwave-dried (E), hot-air-dried vs. microwave-dried (F).

In the 3D PCA model, PC1, PC2, and PC3 contributed 20.1%, 13.4%, and 12.6% to the variance, with a cumulative variance contribution of 46.1% (Fig. 5G). The reliability and predictive capability of the OPLS-DA model were evaluated based on R2 and Q2 values. The R2 and Q2 values for the OPLS-DA models of the D vs. H (Fig. 5J), the D vs. W (Fig. 5K), and the H vs. W (Fig. 5L) all exceed 0.5, indicating acceptable model fit. The closer these values approach 1, the stronger the predictive capability. Furthermore, within the OPLS-DA models, higher VIP values indicate greater contribution from the compounds. When VIP > 1, the corresponding variable can be defined as a key discriminating variable. Based on the dual criteria of VIP > 1 and single-factor ANOVA (*P* < 0.05), among the 30 compounds jointly screened from the D vs. H (Fig. 6A), L-Tyrosine exhibited the highest VIP value of 1.60; Among the 17 compounds screened from D vs. W (Fig. 6B), chlorophyllide a exhibited the highest VIP value of 1.35; among the 12 compounds screened from H vs. W (Fig. 6C), Pteroyltriglutamic acid showed the highest VIP value of 1.26. These substances are likely the primary volatiles underlying the flavor differences in *S. lomentarius* from three drying methods. Hierarchical clustering revealed similarity patterns among different dried metabolites and distribution patterns of various compounds across treatments. Three groups underwent cross-clustering analysis: D vs. H (Fig. 6D), D vs. W (Fig. 6E), and H vs. W (Fig. 6F). Notably, furan compounds (2-pentyl-furan, 2-ethyl-furan) were identified among the differentially expressed compounds in this study. Few furan compounds have been reported by researchers. 2-ethyl-furan exhibits grassy, fatty, and musty odors and has been detected exclusively in *U. pinnatifida* (Ferraces-Casais et al., 2013). 2-Pentyl-furan primarily forms via the cleavage of linoleic acid's 9-hydroxy peroxide to produce a conjugated diene. This diene reacts with oxygen to form peroxyethylene, which then undergoes alkoxy radical cyclization to yield the final 2-pentyl-furan (Xu et al., 2023). In our research, it was found that the freeze-dried group and the microwave group could better retain 2-pentyl-Furan. This compound imparts caramel, buttery, and baked flavors to plant products. Our study also revealed that uneven microwave dried promotes 2-ethyl-furan formation, corroborating Ma et al.'s (Ma et al., 2024) hypothesis that localized overheating and charring are key drivers of this compound. Saturated acetic acid exhibits pungent and vinegary notes, likely generated through oxidation of its corresponding aldehyde. Similar to prior studies, this research identifies Ionone as the key differential volatile in *S. lomentarius* across different drying methods. This ketone compound has been confirmed as a major contributor to seaweed aroma (Zhu et al., 2022) and influences overall flavor during high-temperature drying by enriching ketone-aldehyde notes. Notably, the conclusion that Ionone is a key odor compound in algae is universal. It has also been identified by multiple studies as the primary odor component in freeze-dried algae products (Nader et al., 2022). Our research found that compared with the drying group and the microwave group, the Ionone in the freeze-dried group was upregulated, and it could better retain Ionone. 6-Methyl-3-heptanone exhibits a camphor-like odor, while Dimethyl trisulfide possesses sulfur and fishy notes. These compounds were also identified in *S. platensis*, another algal species (Jia et al., 2025). Compared with the freeze-dried group and the microwave group, the hot air dried group had more Dimethyl trisulfide, which might have endowed the dried group's sargassum with an unpleasant smell. These findings provide clear insights into the extraction of volatile metabolites and their biomarkers across various drying methods.

### 3.14 Correlation analysis between antioxidant capacity and non-volatile metabolites

**Fig. 7:**
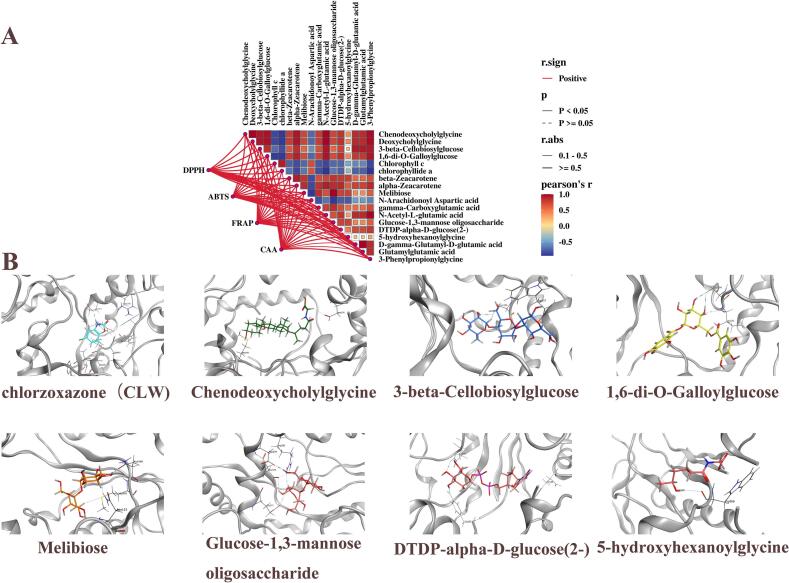
Correlation analysis between four antioxidant activities (DPPH, ABTS, FRAP, and CAA) and differential metabolites (A); Promising 3D binding interactions between the tested compounds and the iNOS-binding pocket compared to CLW (docked)(B).

To analyze the relationship between compounds and antioxidant activity, we selected compounds with significant contributions to biological activity for analysis. Based on correlation coefficients r > ± 0.6 and *P* ≤ 0.05, 18 key differential metabolites were screened. Pearson correlation analysis (r) was performed using antioxidant assays (DPPH, ABTS, FRAP, CAA), as shown in Fig. 7 A. Moreover, 11 compounds were screened out that showed up-regulation in the freeze-dried group but down-regulation in both the hot-air-dried and microwave-dried groups. These compounds were: 1,6-di-O-Galloylglucose, 3-beta-Cellobiosylglucose, alpha-Zeacarotene, beta-Zeacarotene, Chenodeoxycholylglycine, Chlorophyll c, chlorophyllide a, Deoxycholylglycine, DTDP-alpha-d-glucose (2-), Glucose-1,3-mannose oligosaccharide, and Melibiose. Different metabolites exhibited markedly distinct correlation patterns with the four antioxidant assays, indicating that the antioxidant system in *S. lomentarius* is a complex outcome of multiple compounds acting synergistically. Furthermore, different antioxidant methods may target distinct mechanisms of action. All observed metabolites showed significant positive correlations with antioxidant activity (*r* > 0.6) at statistical significance (*P* ≤ 0.05). 1,6-di-O-Galloylglucose, as a hydrolyzable tannin, exhibits strong positive correlations with DPPH, ABTS, and FRAP (*r* > 0.83). Multiple phenolic hydroxyl groups in its structure serve as ideal electron donors, making it one of the primary contributors to the aqueous phase antioxidant capacity of *S. lomentarius*, consistent with the classic antioxidant mechanism of polyphenolic compounds (Farhoosh & Nystrom, 2018). Beta-zeacarotene and alpha-zeacarotene exhibited extremely strong positive correlations with all four antioxidant indicators. This indicates that they are the most critical liposoluble antioxidants in *S. lomentarius*, acting both by directly donating electrons to scavenge free radicals and by possessing strong reducing power. More importantly, they maintain high activity in cellular antioxidant models, suggesting good cellular permeability or bioavailability. Carotenoids possess antioxidant properties, enhance human immunity, and hold potential for preventing certain diseases (F. Wang et al., 2024). Chenodeoxycholylglycine exhibits extremely strong positive correlations with DPPH and ABTS (*r* > 0.92), but shows relatively weaker correlation with CAA (*r* > 0.66). This discrepancy suggests that while they may function as effective radical scavengers in vitro chemical systems, their intracellular bioactivity or absorption efficiency may be limited. Glycine exhibits certain antioxidant effects in vivo by protecting pancreatic β-cells from diabetes-related oxidative stress damage (Chen et al., 2018). This study demonstrates glycine possesses antioxidant capacity, showing a significant positive correlation that is statistically significant. Chlorophyll c and chlorophyllide exhibited strong correlations with DPPH and ABTS, but only moderate correlations with FRAP and CAA. This indicates they excel at directly quenching specific free radical species while being relatively weaker in providing reducing equivalents and intracellular antioxidant efficiency. Chlorophyll influences the antioxidant capacity of algal plants, consistent with our findings. Alpha-zeacarotene and alpha-zeacarotene were identified as the most critical markers of liposoluble and water-soluble antioxidant capacity in *S. lomentarius*, respectively. In summary, the antioxidant capacity of *S. lomentarius* does not rely on a single substance but results from the combined contribution of a complex metabolic network encompassing carotenoids, phenolic compounds, specialized amino acid derivatives, sugars, and pigments.

### 3.15 Docking research

To further explore the mechanism underlying the anti-inflammatory activity of differential metabolites, the present study selected specific target compounds from the key differential metabolites identified in our preliminary work for molecular docking experiments. The candidate compounds were restricted to those that exhibited a marked decrease in abundance in the hot-air-dried group (H) and microwave-dried group (W) compared with the freeze-dried group (D). Previous studies have confirmed that glutamic acid 371 (Glu371), methionine 368 (Met368) and tryptophan 366 (Trp366) are the core amino acid residues in the active pocket of inducible nitric oxide synthase (iNOS), and they play a crucial regulatory role in the binding affinity and inhibitory function of its antagonists (Sun et al., 2026).

The positive control drug chlorzoxazone (CLW) exhibited a binding energy of −4.10 Kcal/mol and an RMSD value of 0.93. It forms a stable hydrogen bond with Glu371 - a core amino acid residue within the iNOS active pocket, which also serves as a key functional site in the enzyme's catalytic domain. Meanwhile, the CLW molecule interacts with the aromatic rings of adjacent residues such as Tyr367 and Tyr341 through π-π stacking. Additionally, hydrophobic residues including valine 346 (Val346) and proline 344 (Pro344) form close contacts with the ligand via hydrophobic interactions, while acidic residues like Asp376 contribute weak polar interactions. The synergistic effect of these multiple intermolecular forces collectively stabilizes the ligand-receptor binding conformation. This interaction pattern is fully consistent with the CLW-mediated iNOS inhibition mechanism reported in existing literature, further validating the accuracy of the docking model established in this study. Based on these findings, the inhibitory potential of candidate anti-inflammatory compounds from *S. lomentarius* can be further evaluated.

Through analyzing the docking scores, root-mean-square deviation (RMSD) values, and binding modes of the 10 screened compounds with the iNOS receptor, seven of them were ultimately identified as potential iNOS inhibitors (Table S8). The three-dimensional interactions between these compounds and the amino acids in the active pocket of iNOS are illustrated in Fig. 7B, while their three-dimensional positioning within the iNOS active pocket and corresponding two-dimensional binding diagrams are detailed in Table S9. Previous studies have confirmed that receptor-ligand interactions rely on the synergy of multiple intermolecular forces, including H-bonds, π bonds, electrostatic interactions, van der Waals forces, and hydrophobic interactions. Among these, hydrogen bonding has been identified as one of the most prevalent and critical interaction modes (Mirzaei et al., 2018). Specifically, chenodeoxycholylglycine exhibited a binding energy of −7.68 kcal/mol and an RMSD value of 1.80, forming two H-bonds with the key residue methionine 349 (Met349) and one with Asp378. For 3-beta-cellobiosylglucose, its binding energy was −7.72 kcal/mol with an RMSD of 0.95, and it established two H-bonds with cysteine 194 (Cys194). Meanwhile, aromatic ring-containing residues such as Trp457 surrounding the ligand formed π-π stacking interactions with the ligand structure, further enhancing its stability within the binding pocket. 1,6-di-O-Galloylglucose showed a binding energy of −6.85 kcal/mol and an RMSD of 1.70, forming two H-bonds with Cys194 and one with Ile. Additionally, hydrophobic interactions were observed as hydrophobic residues (e.g., Val, Pro) approached the ligand - a phenomenon consistent with previous findings that peptide chains rich in hydrophobic amino acid residues tend to enhance anti-inflammatory activity (Lee et al., 2024). Melibiose had a binding energy of −5.76 kcal/mol and an RMSD of 1.71, forming two H-bonds with Asn348 and one with Met349, along with additional π-π stacking and hydrophobic interactions. Glucose-1,3-mannose oligosaccharide displayed a binding energy of −5.23 kcal/mol and an RMSD of 1.52, establishing one hydrogen bond each with Asp376 and Glu371, plus two with Arg382. DTDP-alpha-d-glucose (2-) showed the strongest binding affinity with a binding energy of −8.40 kcal/mol and an RMSD of 1.92; it formed one hydrogen bond each with Gln257 and Tyr367, as well as π-π stacking with the aromatic rings of residues such as Trp188 and Trp340. Finally, 5-hydroxyhexanoylglycine exhibited a binding energy of −5.21 kcal/mol and an RMSD of 0.76, forming one hydrogen bond with Trp366.

The docking results revealed that compared with the reference control CLW, several compounds from *S. lomentarius* samples subjected to drying treatments exhibited favorable binding affinity to iNOS. This suggests that these compounds may possess inherent potential as iNOS inhibitors. Notably, the content of these bioactive candidates decreased significantly during hot-dried and microwave-dried processes. Based on the findings of this study, the impact of drying methods on the metabolite profile of medicinal products should be taken into account when evaluating their pharmaceutical value. However, while molecular docking offers an efficient and rapid approach to predict binding interactions between small molecules and target proteins, it does have certain limitations. Conventional scoring functions often fail to accurately predict the binding affinity of small, highly polar ligands, as the contributions of solvent and entropy are not fully characterized in most docking algorithms (Huang & Zou, 2010).

## 4 Conclusion

This study demonstrated that different drying methods exert distinct effects on the chemical composition and bioactivity of *Scytosiphon lomentarius*. Freeze-dried yielded samples with superior rehydration capacity and higher moisture content. Untargeted metabolomics identified metabolites that directly influence the antioxidant properties of *S. lomentarius*, particularly those categorized into lipids and lipid-like molecules, organic acids and derivatives, and organic oxygen compounds. Molecular docking results revealed that potential bioactive compounds, including chenodeoxycholylglycine, 3-beta-cellobiosylglucose, 1,6-di-O-galloylglucose, melibiose, glucose-1,3-mannose oligosaccharide, DTDP-alpha-d-glucose(2-), and 5-hydroxyhexanoylglycine, exhibited comparable binding affinity/docking scores to co-crystallized inhibitors. Further preliminary identification via HS-GC–MS/MS screened differential volatile compounds, including 2-ethylfuran, Ionone, 6-methyl-3-heptanone, and dimethyl trisulfide, which endow *S. lomentarius* with unique odors depending on the drying method. Overall, the results of this study provide important references for the drying of *S. lomentarius*, thereby facilitating the assurance of the safety and efficacy of its processed products.

## CRediT authorship contribution statement

**Haijiao Lin:** Supervision, Formal analysis. **Yang Song:** Validation, Formal analysis, Data curation. **Yuchen Sun:** Methodology, Investigation. **Qingyun He:** Formal analysis, Data curation. **Pu Xu:** Writing – original draft, Methodology. **Chuhan Feng:** Software. **Liya Duo:** Investigation. **Siming Liu:** Project administration. **Binbin Wei:** Writing – review & editing, Funding acquisition. **Yuan Wang:** Validation, Supervision. **Shaowei Yin:** Writing – review & editing.

## Funding

This study was supported by Shenyang city Science and Technology Program (24–213–3-45), 10.13039/501100007300China Medical University Students Innovation and Entrepreneurship Program (S202510159066).

## Declaration of competing interest

The authors declare that they have no known competing financial interests or personal relationships that could have appeared to influence the work reported in this paper.

## Data Availability

Data will be made available on request.
