# Supplementary material for: Integrated multi-omics reveal the mechanisms of antioxidant, anti-inflammatory and aroma enhancement in Scytosiphon lomentarius via drying methods
Source: Food Chem X. 2026 Jul 1;37:104146. doi: 10.1016/j.fochx.2026.104146 (PMC13351751; doi:10.1016/j.fochx.2026.104146)
Supplement: Supplementary file 1 — Supplementary material 1 [file mmc1.docx]

**Integrated multi-omics reveal the mechanisms of antioxidant, anti-inflammatory and aroma enhancement *in Scytosiphon lomentarius* via drying methods**

Haijiao Lin^a,1^, Yang Song ^d,1^ Yuchen Sun^a^, Qingyun He^a^, Pu Xu^a^, Chuhan Feng^a^, Liya Duo^a^, Siming Liu^a^, Binbin Wei^a*^, Yuan Wang^b*^, Shaowei Yin ^c*^

^a^ School of Pharmacy, China Medical University, No.77 Puhe Road, Shenyang 110122, PR China

^b^ Department of Anesthesiology, Shengjing Hospital of China Medical University, No.36 Sanhao Street, Shenyang, 110001, PR China

^c^ Department of Gynecology and Obstetrics, Shengjing Hospital of China Medical University, No.36 Sanhao Street, Shenyang, 110001, PR China

^d^ Department of Oncology, Shengjing Hospital of China Medical University, No.36 Sanhao Street, Shenyang, 110001, PR China

^*^ Corresponding authors

E-mail addresses: wbb3127@163.com (B. Wei), wangyuan8401@163.com (Y. Wang), yinshaowei1984@163.com (S. Yin)

^1^ These authors contributed equally: Haijiao Lin, Yang Song

**Determination of antioxidant activity**

The antioxidant activity of the samples was assessed by DPPH (2,2-diphenyl-1-picrylhydrazyl) radical scavenging activity assay with appropriate modifications. Briefly, 0.5 mL of appropriately diluted sample extract was mixed with 2.5 mL of freshly prepared DPPH radical methanol solution (80 μM). After standing in the dark for 2 h, the absorbance was measured at 515 nm. Trolox was used as a standard for creating calibration curves (20-200 μM). The results of antioxidant activity were expressed as μmol of Trolox equivalent antioxidant capacity per gram of the sample dry weight (μmol TE/g dw).

The ABTS (2,2′-Azino-bis (3-ethylbenzthiazoline-6-sulfonic acid)) assays were performed using a previous method with minor modifications. ABTS working solution was prepared by mixing potassium persulfate (2.45 mmol/L) and ABTS (7 mmol/L) (1:1, v/v) and incubated at room temperature and protected from light for 12-16 h. The working solution was then diluted with distilled water to obtain an absorbance value of 0.70 ± 0.02 at 734 nm. Next, 200 μL of the appropriately diluted sample solution was mixed with 2.8 mL of ABTS working solution. The mixture was then incubated in the dark at 25 °C for 6 min and its absorbance at 734 nm was measured. Trolox was used as a standard for creating the calibration curve (30-300 μM). The results of antioxidant activity were expressed as μmol of Trolox equivalent antioxidant capacity per gram of the sample dry weight (μmol TE/g dw).

The ferric-reducing antioxidant capacity (FRAP) assay was based on a previous method with some modifications. The FRAP solution consisted of 2.5 mL 10 mM TPTZ solution (0.31 g TPTZ dissolved in 100 mL of 40 mM HCl), 2.5 mL FeCl3·6H2O water solution (20 mM) and 25 mL acetate buffer (0.3 M, pH= 3.6). The mixture was heated to 37 °C before use. 200 μL of the appropriately diluted sample solution was mixed with 2.8 mL of FRAP working solution for 30 min at room temperature and its absorbance was measured at 593 nm. Trolox was used as a standard for creating the calibration curve (40-400 μM). The results of antioxidant activity were expressed as μmol of Trolox equivalent antioxidant capacity per gram of the sample dry weight (μmol TE/g dw).

**Determination of TPC**

The Folin-Ciocalteu reagent was freshly prepared as a 1:9 (v/v) dilution. For the assay, 2 mL of the reagent was combined with 1 mL of diluted sample extract, followed by the addition of 2 mL of 75 g/L sodium carbonate solution. After vortexing for 1 min, the mixture was incubated for 40 min at room temperature in the dark. Absorbance at 760 nm was measured.

**Determination of TFC**

In brief, 0.8 mL of diluted sample extract was combined with 0.3 mL of 5% sodium nitrite and incubated for 6 min in the dark. Then, 0.3 mL of 10% aluminum nitrate was introduced, followed by another 6 min of dark incubation. Subsequently, 4 mL of 1 M sodium hydroxide was added, and the total volume was brought to 10 mL using 60% ethanol. After a final 10 min incubation at ambient temperature in the dark, absorbance was recorded at 510 nm with a microplate reader.

**Determination of Cell viability of RAW246.7 cell**

RAW246.7 cell suspension at a concentration of 1 × 105 cells/mL was added to 96-well culture plates at 100 µL per well and incubated for 12 h. Then, the cells were treated with different concentrations of D, H and W (150, 300, 450 and 600 µg/mL), respectively, for 24 h. Next, 10 µL of CCK-8 solution was added to each well and incubated for 1 h in the dark. Finally, the absorbance of the supernatant in each well was measured at 450 nm using a microplate reader (Infinite M Nano+, Tecan, Switzerland). Medium solution of undissolved sample extract was used as a control.

**Determination of NO content**

NO was measured using the Griess assay. RAW246.7 cell suspension (1 × 105 cells/mL) was added to 96-well plates and incubated in an incubator for 12 h. Subsequently, different concentrations (150, 300, 450 µg/mL) of D, H and W were added to the wells and incubated for 4 h. After 4 h of treatment, cells were incubated with 1 µg/mL LPS for 24 h. A total of 100 µL of cell supernatant was collected and mixed with 100 µL Griess reagent (A: 0.1 % N-(1-naphthyl) ethylenediamine dihydrochloride dissolved in ultrapure water; B: 1 % sulfanilamide dissolved in 5 % phosphoric acid), the reaction was carried out in the dark for 15 min at room temperature and then the absorbance was measured at 540 nm. Sodium nitrite (NaNO_2_) was used as a standard curve to calculate the content of NO.

**Determination of RT-QPCR**

RAW246.7 cell suspension (1 × 106 cells/mL) was added to 6- well plates and incubated in an incubator for 12 h. Cells were pretreated with the samples for 4 h, then treated with 1 µg/ mL LPS and incubated in an incubator for 24 h. TRIZOL reagent was added for total RNA isolation. The HiScript® II Q RT SuperMix for qPCR Kit was used for cDNA synthesis according to the manufacturer’s protocol. Real-time quantitative PCR was performed on an ABI 7500 RT-PCR system using TB Green® Fast qPCR Mix Kit. GADPH was used as an internal reference gene to eliminate the effect of differences in the number of cells in different groups.


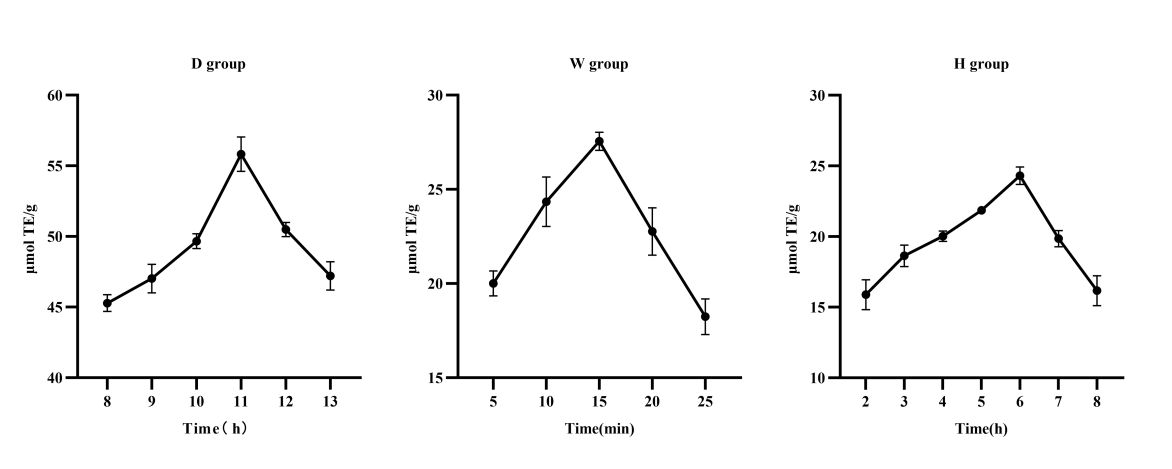


**Fig S1** Optimization of conditions for three drying methods. (D represents vacuum freeze-dried, W represents microwave dried and H represents hot-air dried)


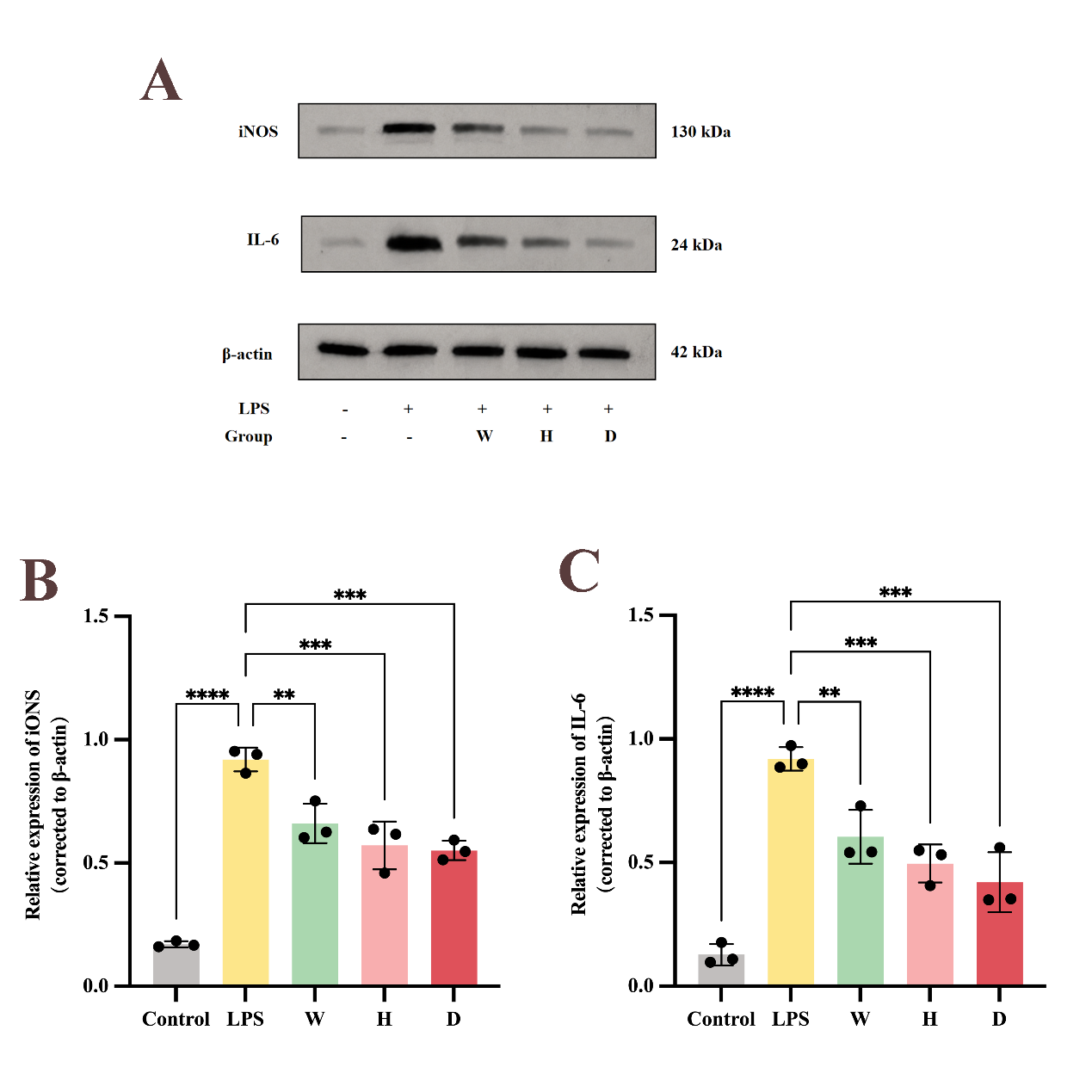


**Fig S2** estern blot analysis of iNOS and IL-6 protein expression bands (A), with β-actin as a loading control. Control: control group; LPS: model group (LPS stimulation alone); Quantitative analysis of iNOS and IL-6 protein expression relative to β-actin by densitometry is shown as bar graphs (B and C). Data are presented as mean ± standard deviation. (* Correlation is significant at the *P*＜0.05; ** Correlation is significant at the *P* < 0.01; *** Correlation is significant at the *P*＜0.001 , **** Correlation is significant at the *P*＜0.0001. D represents vacuum freeze-dried, W represents microwave dried and H represents hot-air dried)


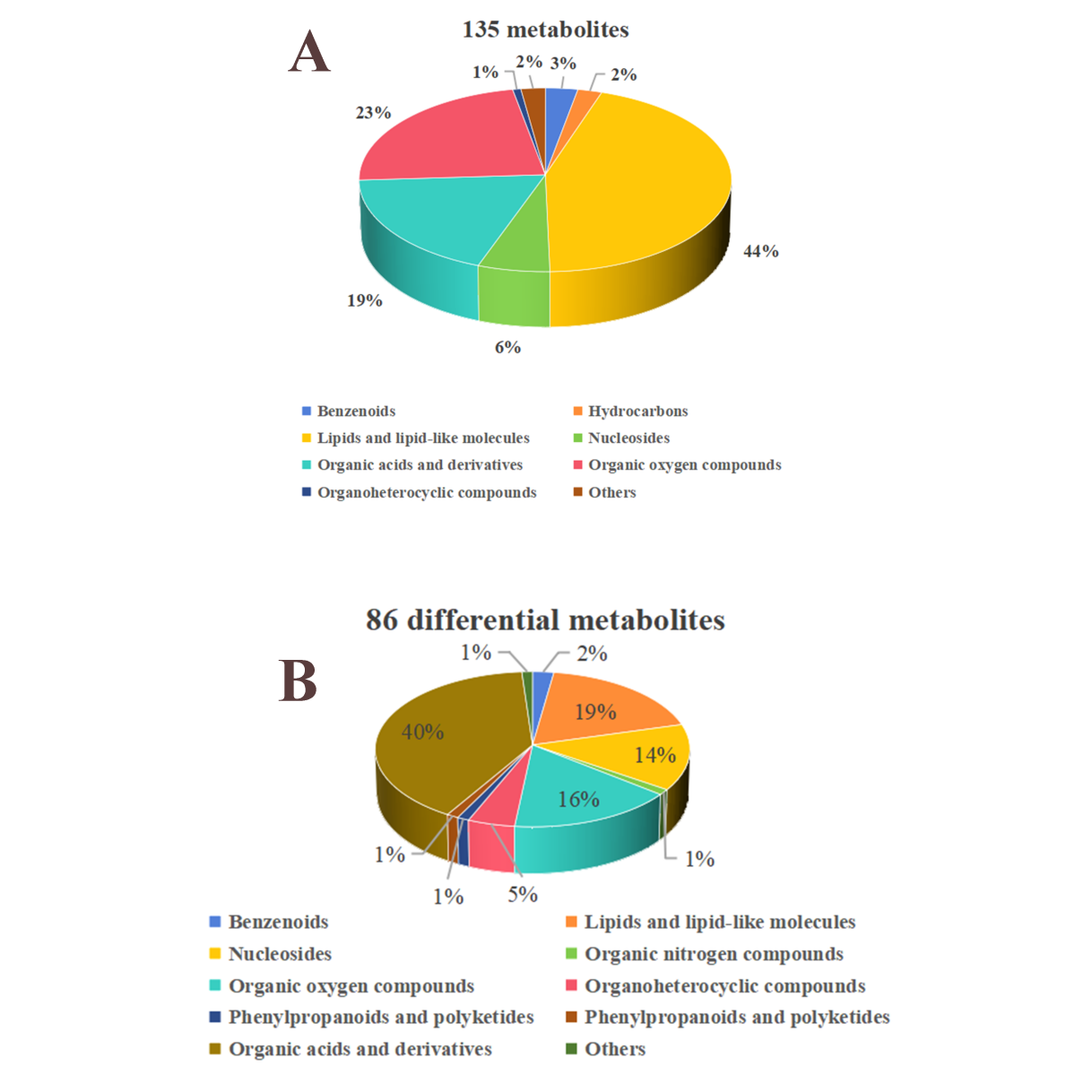


**Fig S3** Super class diagrams of 135 metabolites (A)；Classification diagram of 86 differential Metabolites (B)

**Table. S1 PCR primer sequences**

| Genes | Primer sequence (5’→3’) |
| --- | --- |
| iNOS | (F) 5'-CCCTTCCGAAGTTTCTGGCAGCAG-3' |
|  | (R)5'-GGCTGTCAGAGCCTCGTGGCTTTGG-3' |
| IL-6 | (F) 5'-GTACTCCAGAAGACCAGAGG-3' |
|  | (R) 5'-TGCTGGTGACAACCACGGCC-3' |
| GAPDH | (F) 5'-ACTCACGGCAAATTCAACGGC A-3' |
|  | (R)5'-GACTCCACGACATACTCAGCAC-3' |

**Table. S2 Sensor performance description**

| Array sequence | Sensor name | Performance specification |
| --- | --- | --- |
| 1 | W1C | sensitive to aromatic components, benzene |
| 2 | W5S | highly sensitive to nitrogen oxides |
| 3 | W3C | sensitive to ammonia |
| 4 | W6S | sensitive to hydrides |
| 5 | W5C | sensitive to alkane aromatic components |
| 6 | W1S | sensitive to methane |
| 7 | W1W | sensitive to sulfides |
| 8 | W2S | sensitive to alcohols, aldehydes and ketones |
| 9 | W2W | sensitive to organic sulfides |
| 10 | W3S | sensitive to long chain alkanes |

**Table. S3** In vitro antioxidant activity (DPPH, ABTS and FRAP), cellular antioxidant activity (CAA), total phenolic content (TPC), total flavonoid content (TFC) and antioxidant potency index (ACI) of three drying methods of *S. lomentarius.* D represents vacuum freeze-drying, W represents microwave drying and H represents hot-air drying.

| group | DPPH | ABTS | FRAP | TPC | TFC | CAA | ACI |
| --- | --- | --- | --- | --- | --- | --- | --- |
| D1 | 56.96±0.32a | 58.09±0.13a | 152.75±0.23a | 73.4±0.12a | 77.21±0.7a | 76.92±1.07a | 4.02 |
| D2 | 55.36±0.46b | 56.77±0.25b | 125.6±0.88c | 68.84±0.12c | 74.25±0.34b | 75.3±1.79a | 3.77 |
| D3 | 53.52±0.11c | 55.09±0.61d | 119.16±0.52c | 67.4±0.12d | 71.79±0.72c | 72.76±0.45bc | 3.63 |
| D4 | 53.4±0.17c | 56.03±0.09c | 134.83±6.42b | 70.65±1b | 73.16±1.32bc | 71.77±1.26c | 3.74 |
| D5 | 55.69±1.21b | 57.75±0.15a | 150.03±0.22a | 72.48±0.12a | 76.28±0.75a | 74.91±1.4ab | 3.95 |
| D6 | 55.75±1.04b | 56.96±0.14b | 141.87±9.61b | 71.35±1.1b | 74.32±1.22b | 75.26±1.22a | 3.89 |
| W1 | 29.63±0.47a | 32.32±0.14ab | 88.12±0.38a | 38.21±0.39ab | 40.58±0.26a | 48.7±0.49a | 2.30 |
| W2 | 27.66±0.27c | 31.75±0.59bc | 80.52±0.43d | 36.65±0.09c | 38.38±0.24b | 44.5±1.5d | 2.15 |
| W3 | 28.59±0.09b | 31.55±0.58cd | 85.37±1.14b | 37.64±0.09b | 39.04±1.4b | 47.44±0.16ab | 2.23 |
| W4 | 27.97±0.06bc | 31.8±0.12bc | 82.73±0.06c | 36.85±0.11c | 39.02±0.36b | 46.63±0.54bc | 2.20 |
| W5 | 29.96±0.68a | 32.67±0.56a | 89.03±2.04a | 38.48±0.55a | 40.85±0.62a | 48.9±0.48a | 2.32 |
| W6 | 27.92±0.39c | 30.99±0.08d | 81.5±1.9cd | 36.13±0.79c | 38.37±1.09b | 45.29±1.76cd | 2.16 |
| H1 | 25.59±0.41a | 26.77±0.41a | 61.87±1.45a | 28.02±0.33a | 28.52±0.11a | 29.97±1.1a | 1.80 |
| H2 | 24.83±0.41abc | 26.67±0.65a | 61.02±1.73bc | 27.79±0.14ab | 28.16±0.59a | 28.54±0.32ab | 1.67 |
| H3 | 25.27±0.74ab | 26.51±0.09a | 60.24±0.2abc | 28.04±0.06a | 28.16±0.07a | 29.28±0.9ab | 1.68 |
| H4 | 24.58±0.85bcd | 25.85±0.09b | 59.87±0.33bc | 27.79±0.25ab | 27.7±0.14b | 27.87±1.76bc | 1.64 |
| H5 | 24.04±0.31cd | 26.47±0.16a | 58.69±0.72c | 27.63±0.07bc | 27.37±0.12bc | 27.41±0.5bc | 1.63 |
| H6 | 23.8±0.08d | 25.83±0.09b | 55.75±0.18d | 27.3±0.06d | 27.06±0.09c | 26.36±0.98c | 1.58 |

**Table. S4** Pearson correlation analysis of the vacuum freeze-drying group (D), microwave group (W), and hot-air drying group (H).

|  |  | DPPH | ABTS | FRAP | TPC | TFC | CAA |
| --- | --- | --- | --- | --- | --- | --- | --- |
| D group | DPPH | 1 |  |  |  |  |  |
|  | ABTS | 0.817 | 1 |  |  |  |  |
|  | FRAP | 0.594 | 0.842 | 1 |  |  |  |
|  | TPC | 0.594 | 0.849 | 0.989 | 1 |  |  |
|  | TFC | 0.691 | 0.941 | 0.877 | 0.869 | 1 |  |
|  | CAA | 0.880 | 0.673 | 0.44 | 0.448 | 0.548 | 1 |
|  |  |  |  |  |  |  |  |
| W group | DPPH | 1 |  |  |  |  |  |
|  | ABTS | 0.581 | 1 |  |  |  |  |
|  | FRAP | 0.910 | 0.598 | 1 |  |  |  |
|  | TPC | 0.860 | 0.728 | 0.796 | 1 |  |  |
|  | TFC | 0.725 | 0.804 | 0.822 | 0.721 | 1 |  |
|  | CAA | 0.788 | 0.499 | 0.924 | 0.675 | 0.769 | 1 |
|  |  |  |  |  |  |  |  |
| H group | DPPH | 1 |  |  |  |  |  |
|  | ABTS | 0.419 | 1 |  |  |  |  |
|  | FRAP | 0.666 | 0.692 | 1 |  |  |  |
|  | TPC | 0.683 | 0.657 | 0.835 | 1 |  |  |
|  | TFC | 0.778 | 0.621 | 0.759 | 0.779 | 1 |  |
|  | CAA | 0.523 | 0.401 | 0.617 | 0.54 | 0.653 | 1 |

**Table. S5** Pathway analysis of metabolic changes in vacuum freeze-drying.

|  | Total | Expected | Hits | Raw p | Holm adjust | FDR | Impact |
| --- | --- | --- | --- | --- | --- | --- | --- |
| Purine metabolism | 75 | 0.4944 | 4 | 0.00092367 | 0.084978 | 0.084978 | 0.01062 |
| Porphyrin metabolism | 48 | 0.31641 | 2 | 0.037528 | 1 | 1 | 0.06662 |
| Isoquinoline alkaloid biosynthesis | 6 | 0.039552 | 1 | 0.038969 | 1 | 1 | 0.5 |
| Sulfur metabolism | 12 | 0.079103 | 1 | 0.076566 | 1 | 1 | 0 |
| Tyrosine metabolism | 17 | 0.11206 | 1 | 0.10688 | 1 | 1 | 0.10056 |
| One carbon pool by folate | 21 | 0.13843 | 1 | 0.13048 | 1 | 1 | 0 |
| Phenylalanine, tyrosine and tryptophan biosynthesis | 22 | 0.14502 | 1 | 0.13629 | 1 | 1 | 0.02002 |
| Starch and sucrose metabolism | 22 | 0.14502 | 1 | 0.13629 | 1 | 1 | 0.30032 |
| Galactose metabolism | 27 | 0.17798 | 1 | 0.16483 | 1 | 1 | 0.00553 |
| Glycerophospholipid metabolism | 38 | 0.25049 | 1 | 0.22466 | 1 | 1 | 0.05023 |
| Pyrimidine metabolism | 41 | 0.27027 | 1 | 0.24029 | 1 | 1 | 0.01956 |
| Ubiquinone and other terpenoid-quinone biosynthesis | 48 | 0.31641 | 1 | 0.27567 | 1 | 1 | 0 |

**Table. S6** Pathway analysis of metabolic changes in hot-air drying.

|  | Total | Expected | Hits | Raw p | Holm adjust | FDR | Impact |
| --- | --- | --- | --- | --- | --- | --- | --- |
| Purine metabolism | 75 | 0.59328 | 4 | 0.002018 | 0.18565 | 0.18565 | 0.01062 |
| Pyrimidine metabolism | 41 | 0.32432 | 2 | 0.039638 | 1 | 1 | 0.04885 |
| Porphyrin metabolism | 48 | 0.3797 | 2 | 0.052875 | 1 | 1 | 0.06662 |
| Sulfur metabolism | 12 | 0.094924 | 1 | 0.091218 | 1 | 1 | 0 |
| Arginine biosynthesis | 18 | 0.14239 | 1 | 0.13391 | 1 | 1 | 0.03704 |
| One carbon pool by folate | 21 | 0.16612 | 1 | 0.15455 | 1 | 1 | 0 |
| Biosynthesis of unsaturated fatty acids | 22 | 0.17403 | 1 | 0.16134 | 1 | 1 | 0 |
| Glycerophospholipid metabolism | 38 | 0.30059 | 1 | 0.26328 | 1 | 1 | 0.05023 |
| Carotenoid biosynthesis | 43 | 0.34015 | 1 | 0.29272 | 1 | 1 | 0.01754 |

**Table. S7** Pathway analysis of metabolic changes in microwave drying.

|  | Total | Expected | Hits | Raw p | Holm adjust | FDR | Impact |
| --- | --- | --- | --- | --- | --- | --- | --- |
| Purine metabolism | 75 | 0.64272 | 4 | 0.0028064 | 0.25819 | 0.25819 | 0.01062 |
| Phenylalanine, tyrosine and tryptophan biosynthesis | 22 | 0.18853 | 2 | 0.014222 | 1 | 0.6542 | 0.02152 |
| Isoquinoline alkaloid biosynthesis | 6 | 0.051417 | 1 | 0.05041 | 1 | 1 | 0.5 |
| Porphyrin metabolism | 48 | 0.41134 | 2 | 0.061251 | 1 | 1 | 0.06662 |
| Tropane, piperidine and pyridine alkaloid biosynthesis | 9 | 0.077126 | 1 | 0.074725 | 1 | 1 | 0 |
| Sulfur metabolism | 12 | 0.10283 | 1 | 0.098464 | 1 | 1 | 0 |
| Phenylalanine metabolism | 12 | 0.10283 | 1 | 0.098464 | 1 | 1 | 0.42308 |
| Tyrosine metabolism | 17 | 0.14568 | 1 | 0.13678 | 1 | 1 | 0.10056 |
| Arginine biosynthesis | 18 | 0.15425 | 1 | 0.14427 | 1 | 1 | 0.03704 |
| One carbon pool by folate | 21 | 0.17996 | 1 | 0.16635 | 1 | 1 | 0 |
| Biosynthesis of unsaturated fatty acids | 22 | 0.18853 | 1 | 0.1736 | 1 | 1 | 0 |
| Cyanoamino acid metabolism | 29 | 0.24852 | 1 | 0.2227 | 1 | 1 | 0 |
| Glycerophospholipid metabolism | 38 | 0.32564 | 1 | 0.28188 | 1 | 1 | 0.05023 |
| Pyrimidine metabolism | 41 | 0.35135 | 1 | 0.30066 | 1 | 1 | 0.01956 |
| Phenylpropanoid biosynthesis | 43 | 0.36849 | 1 | 0.31293 | 1 | 1 | 0 |
| Carotenoid biosynthesis | 43 | 0.36849 | 1 | 0.31293 | 1 | 1 | 0.01754 |
| Ubiquinone and other terpenoid-quinone biosynthesis | 48 | 0.41134 | 1 | 0.34274 | 1 | 1 | 0 |
| Glucosinolate biosynthesis | 65 | 0.55702 | 1 | 0.43539 | 1 | 1 | 0 |

**Table S8.** Binding scores and interactions of the selected 17 compounds clustered into the iNOS binding pocket compared to the co-crystallized inhibitor, CLW.

| Compound | S^a^ | RMSD^b^ | Amino acid bond | L^c^ |
| --- | --- | --- | --- | --- |
| CLW | -4.10 | 0.93 | Glu371/H- donor | 2.92 |
| Chenodeoxycholylglycine | -7.68 | 1.80 | Met349/H- donor  Met349/H- donor  Asp376/H-donor | 3.95  3.06  3.41 |
| 3-beta-Cellobiosylglucose | -6.26 | 1.35 | Trp457/H-donor  Cys194/ H-donor  Cys194/ H-donor | 3.46  3.77  3.45 |
| 1,6-di-O-Galloylglucose | -6.85 | 1.70 | Cys194/ H-donor  Cys194/ H-donor  Ile195/H-acceptor | 3.45  3.93  3.16 |
| Chlorophyll c | -8.11 | 1.58 |  |  |
| Melibiose | -5.76 | 1.71 | Asn348/H-donor  Asn348/H-donor  Met349/H-donor  Asn115/H-acceptor | 3.17  3.20  3.85  3.17 |
| Glucose-1,3-mannose oligosaccharide | -5.23 | 1.52 | Glu371/ H-donor  Asp376/ H-donor  Asp379/ H-donor  Arg382/H-acceptor  Arg382/H-acceptor | 3.28  3.14  2.82  3.36  2.93 |
| DTDP-alpha-D-glucose(2-) | -8.40 | 1.29 | Tyr367/ H-donor  Gln257/H-acceptor  Trp188/H-pi | 2.94  3.30  4.29 |
| 5-hydroxyhexanoylglycine | -5.21 | 0.76 | Trp366/ H-donor | 3.32 |

**Table S9.** 3D positioning and 2D binding interactions between the promising tested compounds at the iNOS-binding pocket compared to CLW (docked).

| **Compound** | **3D positioning** | **2D binding interactions** |
| --- | --- | --- |
| CLW | 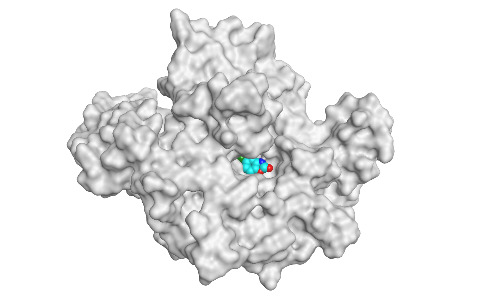 | 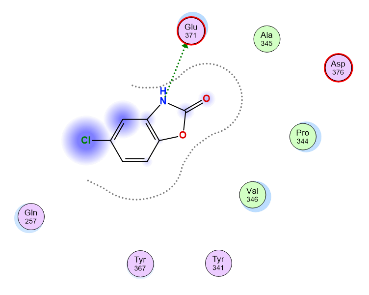 |
| Chenodeoxycholylglycine | 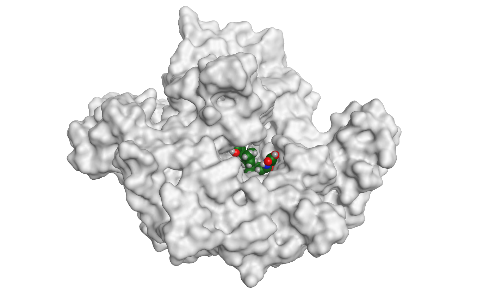 | 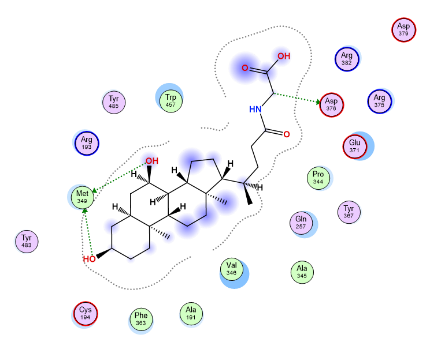 |
| 3-beta-Cellobiosylglucose | 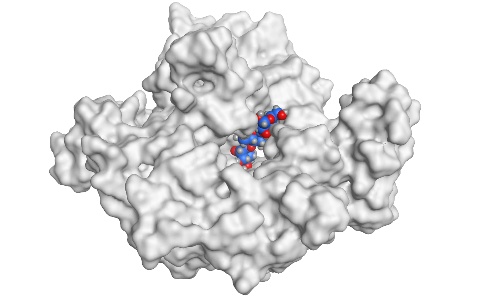 | 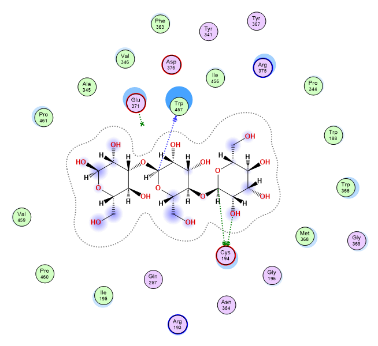 |
| 1,6-di-O-Galloylglucose | 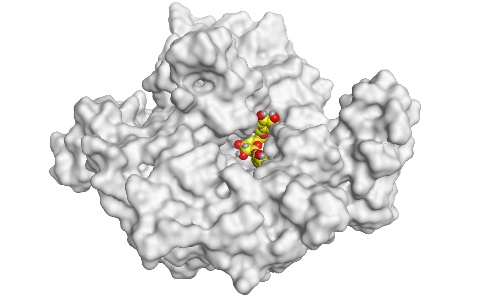 | 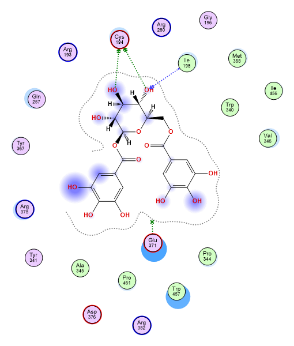 |
| Chlorophyll c | 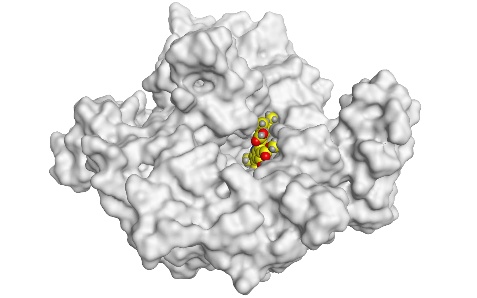 | 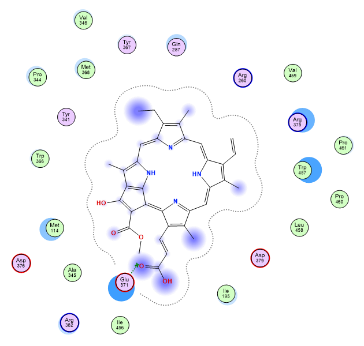 |
| Melibiose | 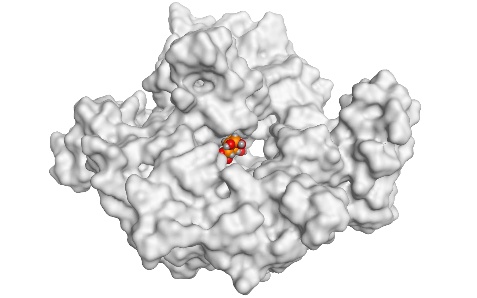 | 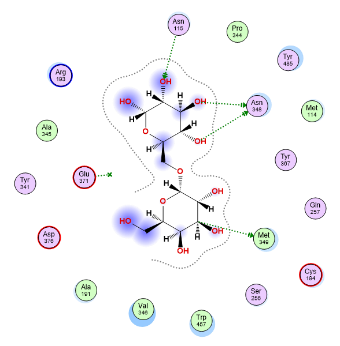 |
| Glucose-1,3-mannose oligosaccharide | 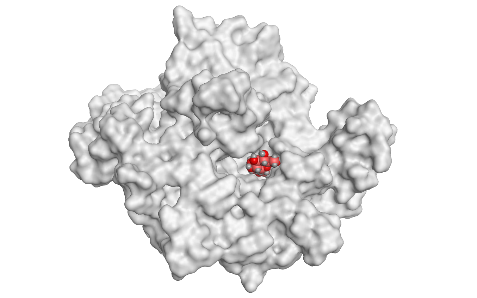 | 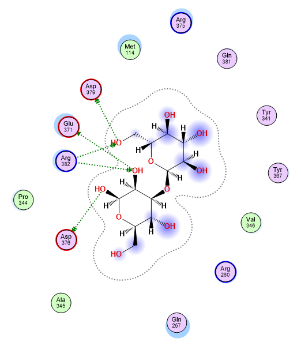 |
| DTDP-alpha-D-glucose(2-) | 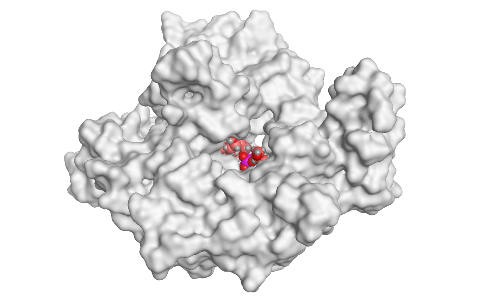 | 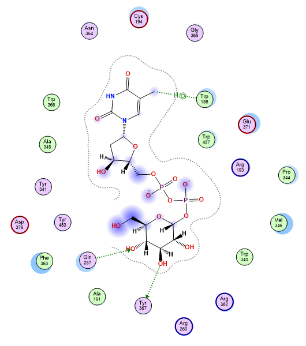 |
| 5-hydroxyhexanoylglycine | 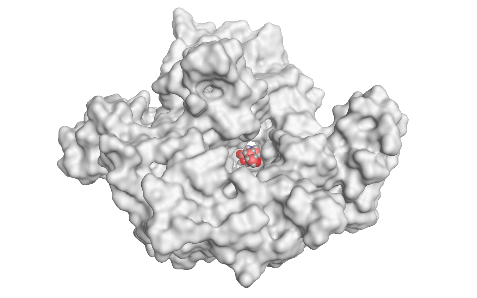 | 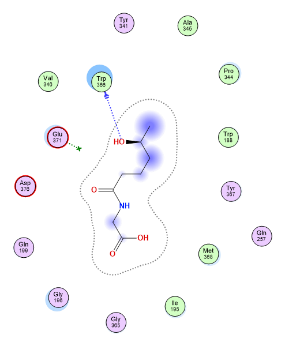 |
